# Smart city lifestyle sensing, big data, geo-analytics and intelligence for smarter public health decision-making in overweight, obesity and type 2 diabetes prevention: the research we should be doing

**DOI:** 10.1186/s12942-021-00266-0

**Published:** 2021-03-03

**Authors:** Maged N. Kamel Boulos, Keumseok Koh

**Affiliations:** 1 School of Information Management, Sun Yat-Sen University, East Campus, Guangzhou, 510006 Guangdong China; 2 Department of Geography, The University of Hong Kong, Pokfulam RD, Hong Kong, China

**Keywords:** Smart city lifestyle sensing, Big data, Geo-analytics, Systems science, Overweight, Obesity, Type 2 diabetes

## Abstract

The public health burden caused by overweight, obesity (OO) and type-2 diabetes (T2D) is very significant and continues to rise worldwide. The causation of OO and T2D is complex and highly multifactorial rather than a mere energy intake (food) and expenditure (exercise) imbalance. But previous research into food and physical activity (PA) neighbourhood environments has mainly focused on associating body mass index (BMI) with proximity to stores selling fresh fruits and vegetables or fast food restaurants and takeaways, or with neighbourhood walkability factors and access to green spaces or public gym facilities, making largely naive, crude and inconsistent assumptions and conclusions that are far from the spirit of 'precision and accuracy public health'. Different people and population groups respond differently to the same food and PA environments, due to a myriad of unique individual and population group factors (genetic/epigenetic, metabolic, dietary and lifestyle habits, health literacy profiles, screen viewing times, stress levels, sleep patterns, environmental air and noise pollution levels, etc.) and their complex interplays with each other and with local food and PA settings. Furthermore, the same food store or fast food outlet can often sell or serve both healthy and non-healthy options/portions, so a simple binary classification into 'good' or 'bad' store/outlet should be avoided. Moreover, appropriate physical exercise, whilst essential for good health and disease prevention, is not very effective for weight maintenance or loss (especially when solely relied upon), and cannot offset the effects of a bad diet. The research we should be doing in the third decade of the twenty-first century should use a systems thinking approach, helped by recent advances in sensors, big data and related technologies, to investigate and consider all these factors in our quest to design better targeted and more effective public health interventions for OO and T2D control and prevention.

**Supplementary Information:**

The online version contains supplementary material available at 10.1186/s12942-021-00266-0.

## Introduction

The ongoing pandemic of coronavirus disease (COVID-19) has heavily scarred the healthcare capacities and systems across the world during the whole year of 2020 and until now (Q1 2021). At the time of writing, more than 1 per cent of the world population (82 million) were infected (diagnosed/confirmed cases only), and over 1.8 million deaths were claimed by the pandemic [1]. Despite the recent arrivals of vaccines and medications, COVID-19 is likely to pose an additional burden to public health for the next few years [2]. While an effective control and prevention of COVID-19 would be high on the list of public health interventions worldwide, combating the existing global epidemic of non-communicable diseases (NCDs), attributable each year for 41 million deaths or 71 per cent of all death globally, should not be neglected [3].

Among NCDs, overweight and obesity (OO) and type 2 diabetes (T2D) are unique because they are both medical conditions (OO: ICD-10 code E66; T2D: ICD-10 code E11) and leading risk factors for other comorbidities, including many NCDs and infectious diseases such as COVID-19 [4–7]. The impacts of OO and T2D on public health are much devastating. A global study estimated that about 9.7 per cent of the world population (711.4 million) were obese and 4.0 million deaths were attributable for obesity in 2015 [8]. T2D affected 463 million adults aged 20 to 79 years worldwide and caused 4.2 million deaths in 2019 [9]. Scholars predicted no decline in OO and T2D prevalence any time soon if the current increasing trend continues [10, 11].

The fact that OO and T2D are unevenly distributed across different socioeconomic statuses (SES), demographic groups and geographies make researchers and policymakers more puzzled to design effective public health interventions [12, 13]. Low-SES individuals, specific racial/ethnic groups (e.g., OO: non-Hispanic blacks in the United States and Western Pacific Islanders; T2D: non-Hispanic black, Hispanic-American and Native American population in the United States, and South Asian and black African/Caribbean population in the United Kingdom), and rural populations were found more vulnerable to OO and/or T2D [14–17]. Notably, China and India, the two most populous and emerging economies in the world, are among the world's largest and fastest-growing populations with OO and/or T2D [18, 19]. Designing effective prevention and control policies for OO and T2D is of importance not only to decrease their direct epidemiological costs, but also to ensure successful achievement of the United Nation's Sustainable Development Goals (SDGs) [20].

To get our act together for successful future strategies and interventions, this article provides a brief overview of the latest OO and T2D geospatial research trends, especially focusing on novel approaches using precision and accuracy medicine/public health, smart technologies, big data and systems science.

## Innovations in research methods during the last decade

OO and T2D have been conventionally and rather naively attributed to a long-term energy imbalance between calories intake from food and beverages and expenditure via physical activity (PA) and behaviour, but the reality of their pathogenesis is far more complex than that. Researchers have extensively investigated the multifactorial and deeply interrelated interactions among multiple biological, sociodemographic and contextual factors of OO and T2D. Clinic-based studies have identified more than 50 genes associated with OO and 36 genes related to T2D that play specific roles in appetite and food intake, insulin action, cholesterol and fatty acid synthesis, and family heredity [21, 22]. Population-based studies have also found various risk factors, including, but not limited to, age, race/ethnicity, SES, lifestyle choices and behaviours, sugar and high-fructose corn syrup-laden food products (not all calories are equal in their health effect, even though each of them carries the same amount of energy), and different environments [23].

Geographic Information Systems (GIS) have contributed uniquely to OO and T2D literature. Researchers used GIS to investigate the built environment and its associations with OO and T2D. Since 2002 (and up to December 2020), more than 130 articles using GIS have been published in *International Journal of Health Geographics* (IJHG) on the topic of OO (n = 77) and T2D (n = 64). Examples of OO and T2D articles published in IJHG include studies about youth's obesity [24, 25], older people's body weight [26], the built environment [27, 28] and new methodologies [29, 30]. With advances in related technologies, traditional GIS research approaches have become smarter. Geo-analytics and GeoAI are two rapidly growing GIS research areas with promising applications in the formulation and monitoring of OO and T2D public health interventions. Geo-analytics combines location-based (big) data with advanced analysis functionalities on a website or application (desktop or mobile), enabling interactive data interrogation, analysis and visualisation. GeoAI covers a range of technologies for extracting meaningful information and knowledge from geospatial big data using artificial intelligence and data science methods, such as data mining, machine learning/deep learning and high-performance computing [31, 32].

During the past few years, new technologies have been applied in OO and T2D research. Firstly, utilising lifestyle sensors (LS) has gained popularity among researchers. LS are devices that detect and collect changes in the environment or behaviours of an object and relay the collected data to other data processing devices or controllers (e.g., smartphones and/or cloud computing platforms). Types and levels (intensity and duration) of physical activity, food intake, mobile personal indirect calorimetry, heart rate (variability), blood sugar, blood lipids and blood pressure levels are among the measures that OO and T2D studies have frequently used [33] or can use (see, for example, slide 30 in Additional file 1). Secondly, big data have proved their potential to enhance our understanding of OO and T2D. Data aggregates from restaurant chains, supermarkets and their loyalty card schemes/apps (e.g., about sales and customers' purchasing habits/patterns), on-board vehicle sensors, weight management programmes (e.g., clinical and sociodemographic data from clients), geospatial built environment (e.g., layouts of restaurants and the different types of food they serve, remote sensing data, etc.), social media, and smartphones and wearables (e.g., fitness apps and smartwatches) have all been (or can be) collected, crosslinked and utilised for advanced analyses, e.g., [34], subject to appropriate individual privacy safeguards. And finally, systems science—an interdisciplinary field of science to investigate highly complex structures and interactions among components within systems—have helped researchers adopt a holistic understanding of OO and T2D, especially focusing on lifestyle, food environment, social networks (offline and online), and healthcare [35–39].

## What we need to do in future OO and T2D studies: a feasibility demonstrator roadmap for a large metropolis

This demonstrator (see Additional file 1) features Guangzhou, the capital and most populous city (total population: about 15 million in 2019) of Guangdong Province in China, as the study area, but the same roadmap can be adopted for other cities/regions in China and other countries. As of 2020, Guangzhou is the 4th largest regional economy by Gross Domestic Product in China (1.7 trillion Chinese yuan, equivalent to 262 billion US dollars). With its very rapid economic development during the last few decades, China is now confronting the dual epidemics of OO and T2D. It has the largest overweight population in the world, and its population figures for childhood obesity and adult obesity ranked 1st and 2nd, respectively, across the world in 2014/15 [8, 40]. Over one out of ten Chinese adults (10.9%) were estimated to have diabetes in 2013 [41]. Immediate, evidence-based public health interventions focusing both on locale- and population-specific evidence will be vital to help release China from the tightening grip of OO and T2D.

Previous research into food and PA neighbourhood environments has mainly attempted to associate body mass index (BMI) with proximity to stores selling fresh fruits and vegetables or fast food restaurants and takeaways, or with urbanisation, neighbourhood walkability factors and access to green spaces or public gym facilities, making largely naive, blanket assumptions and crude, incomplete and often inconsistent (across similar studies) conclusions that are far from the spirit and requirements of twenty-first century precision and accuracy public health.

Different people and population groups respond differently to the same food and PA environments, due to a myriad of unique individual and population group factors and their complex interplays with each other and with food and PA elements (genetic/epigenetic factors, metabolic factors, gut bacteria profiles, gut hormones profiles, health literacy profiles, dietary and lifestyle habits, screen viewing times, stress levels, sleep patterns, SES, local cuisine and food industry standards and regulations [e.g., food processing levels, food labelling practices, etc.], environmental air and noise pollution levels, activity spaces [associating people with a single address/postcode is not always ideal in food and PA environment studies], etc.).

Furthermore, the same food store or fast food outlet can often sell or serve both healthy and non-healthy options/portions and use both good and bad food processing and cooking methods for different products, so a simple binary classification into 'good' or 'bad' store/outlet should be avoided. Population dietary behaviours, including amounts consumed per individual snack/meal/day or per family are also important, as even the healthiest options can prove unhealthy when overconsumed. Moreover, appropriate physical exercise, whilst essential for good health and disease prevention, is not very effective for weight maintenance or loss (especially when solely relied upon), and cannot offset the effects of a bad diet [42]. In fact, the "wrong" type of physical exercise might sometimes result in weight gain in certain individuals, and research has shown that some individuals avoid or 'hate' exercise because of their genetic makeup, even when living in close proximity to green spaces and public gyms. As far as urbanisation is concerned, research also shows the gap of BMI between urban and rural is closing, mostly by an unprecedented increase in rural BMI around the world in recent years, especially in low- and middle-income regions (so it is not urbanisation that is to blame as such or alone) [43].

**Fig. 1:**
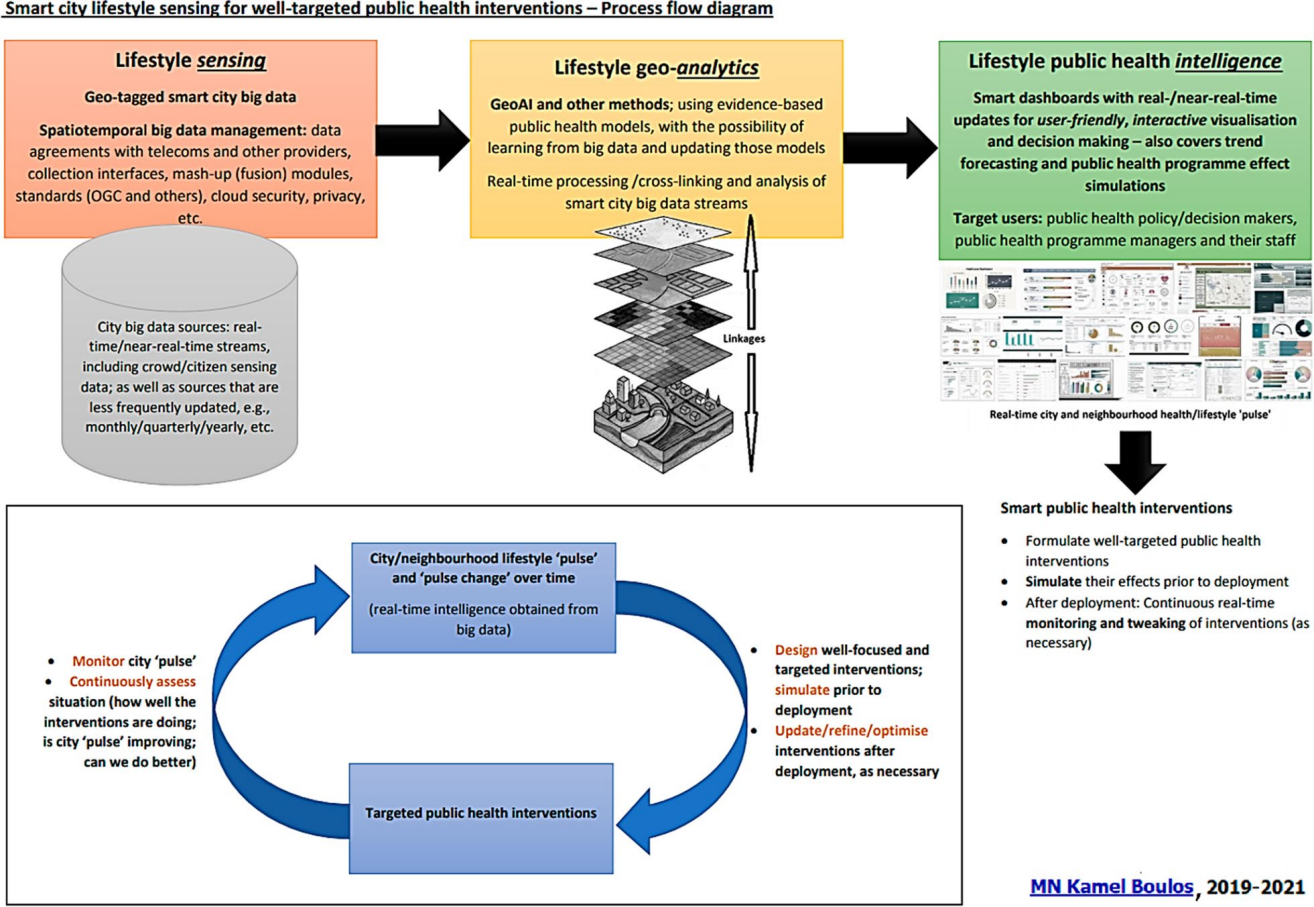
Process flow diagram of the proposed research demonstrator roadmap

The research we should be doing in the third decade of the twenty-first century should use a systems thinking approach, helped by recent advances in sensors, big data and related technologies, to cater for this myriad of interconnected factors in our quest to design better targeted and more effective public health interventions for OO and T2D control and prevention. The remainder of this section briefly describes the high-level task clusters that are necessary to successfully execute the feasibility demonstrator roadmap and build prototype smart dashboards for future precision and accuracy public health OO and T2D interventions (Fig. 1).

### Task cluster 1: Agree a comprehensive list of key population lifestyle data

To design and develop effective evidence-based public health interventions for OO and T2D, there should be an agreement on a comprehensive list of population lifestyle data relevant to the study area and study population. Besides standard population variables, such as age, gender, race/ethnicity, SES and family status, a set of important lifestyle risk factors for OO and T2D studies may include health literacy levels (per population group/neighbourhood derived from up-to-date, representative community-wide surveys), food shopping and dietary habits, behavioural characteristics (e.g., sedentary lifestyle, physical activity, screen viewing, sleep, smoking, mental health, etc.), relevant health conditions and exposure to environmental pollutants (e.g., noise, light pollution and fine particulate matter).

#### Population dietary habits

While studies have often requested participants to write food diaries (usually resulting in inaccurate and/or incomplete data collection), later studies have used dedicated smartphone apps to collect and analyse meal records and food barcodes (packaged products) and pictures (using machine learning and computer vision, sometimes coupled with smartphone spectroscopy and other sensors [44]). Aggregates of individual-level dietary habit data from specialised smartphone apps (see, for example, slide 30 in Additional file 1) may be further combined with territory-wide databases of food outlets, food/restaurant guide and review websites, food sales/payment transaction records (segmented by food class/type), food retailers consumer loyalty card/app data aggregates (can provide detailed geodemographic classifications of households and consumers, offering unique insights about food spending patterns/trends among different population groups), purchasable food options/availability, and/or other relevant food databases at various neighbourhood levels.

#### Population PA and behavioural characteristics

Measures of PA and estimates of energy expenditure levels, e.g., by computing daily walking steps, are commonly used in OO and T2D research. Some studies measured calories spent on PA using LS, GPS/location-based applications or exergaming devices [45, 46]. Fitness wearables and apps also exist that can perform some form of PA segmentation by type and intensity, which when combined with specialised LS for mobile personal indirect calorimetry, can further calibrate and enhance the accuracy of energy expenditure estimates [42]. Big data aggregates from these wearables, gadgets and apps can reveal interesting trends among different population groups.

Other measurable lifestyle factors associated with PA in OO and T2D literature include the use of television and other screen devices (can affect sleep quality), sugar-sweetened beverage intake (often marketed using football and sport themes, which can be deceiving), smoking (reduces physical endurance) and sleep quality [47, 48]. Smartphone screen time trends, sedentary behaviour/walking steps and sleep quality/patterns in the community can be readily extracted from user population big data aggregates derived from smartphones (e.g., Google Digital Wellbeing platform on Android) and fitness/PA apps and gadgets, providing invaluable 'lifestyle intelligence' for designing better public health interventions.

Further population-level PA indicators to consider include population heart rate variability levels, also derived from fitness/PA wearable and app data aggregates; and, if a platform such as Google Fit is used, average 'Move Minutes' and 'Heart Points' per person/per day in different age groups and country regions, cities and city neighbourhoods; percentages of poor, average and high performers in target populations; also cumulative (total) population walking steps, 'Move Minutes' and 'Heart Points' per region, city and neighbourhood for a given period. These indicators can be monitored and compared every few months to determine population progress and trends over time, or at different times of the year/seasons, or following specific PA community interventions. Indicators can also be normalised by population number to compare different regions, cities and neighbourhoods. (Location-tagging of app data aggregates, e.g., by neighbourhood or city, should be straightforward and readily available, as most PA and fitness mobile apps require 'location permission' to log user's home base location, fetch corresponding weather data, and map walking, running and cycling routes.) Further interesting patterns might emerge from user data aggregates that can help city planners better understand and optimise the PA landscape of their city and its population; for example, data might reveal that residents of some neighbourhoods are doing most of their daily exercises in some walkable streets, parks or public gym facilities in a different neighbourhood [42].

#### Population health status/conditions and stress levels

Population clinical data (aggregates and trends stratified by population group and neighbourhood/region), such as BMI and body fat percentage (BFP, a better measure than just body weight or BMI), blood sugar and blood pressure levels, cholesterol/blood lipid profiles, data about relevant long-term conditions, etc., can be combined with other population lifestyle factors/data, since OO and T2D are related to, or often coexist with, other chronic diseases, e.g., cardiovascular conditions.

Population aggregates from platforms such as Amazon's Halo wearable and health monitoring service (subscription) can provide unique insights about the prevailing moods and stress levels among different population groups over time. Powered by machine learning, speech processing and computer vision, Amazon Halo infers mood and stress levels from the wearer's voice tone, and also measures BFP [49].

#### Activity spaces, exposure to environmental pollutants, etc.

The rapid advances and improvements in mobile technologies and their affordability over the past two decades have enabled researchers to use smart devices and wearables more actively in their research. For example, a Dutch study obtained a GPS-tracked mobility dataset from its participants for seven consecutive days [50]. Such a dataset, if collected for a longer period of months or years, could be extremely useful for researchers to measure the precise and accurate levels of exposure to environmental factors, rather than using a home, school, or office address as a proxy for exposure locations, (in addition to providing more accurate and highly valuable details about participants' *actual* food and PA environments). Citizen noise pollution monitoring is also possible using smartphones [51]. The analysis of relevant photos and videos of neighbourhood environments and street scenes can provide additional insights.

It should be noted that the aforementioned lifestyle variables are not an exhaustive list of all the factors one should consider. Researchers also need to prioritise the acquisition of these variables according to their relative importance, data availability and reliability, and ease and cost of collection, by carefully examining previous public health interventions (their level of success and types of data feeding into them), the decision support needs of public health professionals and policymakers, and the available human and financial resources.

### Task cluster 2: identify the most efficient ways of data collection

Identifying the most efficient, appropriate and reliable ways of data collection is as important as selecting the relevant variables. Technological feasibility, as well as traditional issues, such as budget and staffing, should all be examined when planning data collection. There are several important questions that researchers need to carefully consider; for example, are the data purchasable from vendors or need to be collected? Will the data be collected by a research team, by some outsourcing contract, or by crowdsourcing? Which method or instrument would be best for collecting each dataset depending on its nature, real- or near-real-time datasets and those that need to be collected less frequently; for example, using data streams from LS/wearables, smartphone apps, or traditional survey methods (e.g., by telephone, post, websites, or social media)? What levels of detail granularity will be set for the research and data (e.g., by some specified population groups, neighbourhood, or city)? How long will it take to collect enough data for the system to reason with, and what will be the ideal update frequency/interval, where applicable (e.g., (every) 24 h, one week, one month, yearly, or before, during and after some public health intervention, etc.)? For data that are going to be sampled rather than extracted from population aggregates of routine service/app users, we need to determine how many participants should be recruited (and their characteristics) to secure a representative sample of the target population, and what is the most appropriate method to recruit them (e.g., snowball sampling, convenience sampling, stratified sampling, etc.)? (Big) data quality and reliability dimensions must also be carefully considered for each dataset, e.g., validity, accuracy, consistency, completeness, timeliness, etc.

### Task cluster 3: data warehousing and management

This task cluster focuses on big data governance and management, covering a number of interrelated issues that are critical to the success, scalability and sustainability of the demonstrator, including compliance with relevant data and metadata standards, e.g., OGC (Open Geospatial Consortium) standards; compliance with data protection regulations, ensuring individual data privacy is always preserved, especially when non-aggregate data are collected and processed; establishing adequate data sharing agreements with providers; and the adoption of best practices in ontologies, big data warehousing and cloud security.

Specifically, data should be protected from any unauthorised access and potential corruption at all times. Individuals' privacy and confidentiality protection should comply with up-to-date regulations for data storage and management, all while enabling the demonstrator's seamless, secure access to the data for analysis. Data taxonomies and ontologies are particularly essential for managing and reasoning with big data, since the majority of big data are often collected in unstructured form [52, 53].

### Task cluster 4: data fusion, geo-analytics and GeoAI

Data fusion refers to the process of integrating data from different datasets and multiple sources, including managing data uncertainties and incompleteness/missing data points across the fused datasets. For example, a geo-tagged social media dataset aggregated at neighbourhood levels can be 'fused' with other aggregate neighbourhood data, such as governmental census or survey data, to paint a more accurate and detailed picture of the target population. Geo-analytics and GeoAI tools are becoming increasingly essential for crosslinking and reasoning with ('making sense of') the growing amounts of geo-tagged population big data that are continually generated through mobile health (mHealth) devices/apps and precision medicine practices [32].

### Task cluster 5: design smart public health dashboards with mechanisms for intervention simulations and near-real-time monitoring and optimisation of interventions.

The smart public health dashboards should (1) provide public health decision makers and programme planners with a user-friendly one-stop portal for intelligent data analysis, interactive data querying and visualisation in different ways, and distributed team collaboration; and (2) offer lay summaries/views of the dashboards to keep members of the general public informed and engaged. Public health planners can use the dashboards and the generated big data intelligence to interactively design and prioritise new public health interventions, identify their potential target population groups and areas, and perform simulation modelling of intervention costs and impacts under different settings or 'what if' scenarios prior to deployment [42]. Various GIS and simulation modelling techniques are available for this purpose (see one example in [37]). Once an intervention is agreed and deployed in the real world, the smart dashboards can then serve as a 'situation room'. Fed with fresh, post-intervention-deployment data streams, the dashboards can be used to dynamically monitor the intervention in near real-time and tweak it as necessary. The ideal dashboards should be intuitive and user-error-tolerant. They should help decision makers uncover subtle, unfolding changes of concern among their populations before they grow into bigger problems. To achieve this goal, the dashboards should serve intelligent, contextual alerts and reminders to its users in a timely manner, inferred from the combined 'patterns of change' of multiple interrelated data streams, (going beyond conventional, simple threshold-based triggers derived from single data sources, which often fail to provide timely 'early warnings').

### Task cluster 6: evaluate dashboards and other components

Public health dashboards serve critical functions as information and intelligence communication tools and in decision support. Adequate user involvement in all stages of their development (via a representative sample of all stakeholders and end users' roles) is key to their lasting success and ultimate user acceptance [54]. Development should be conducted iteratively and incrementally to incorporate user feedback. A comprehensive, multi-faceted evaluation, covering usability, utility, accuracy/reliability, etc., must be carried out for all the interfaces, tools and other components created in task clusters 3, 4 and 5 above, individually and in their integrated form as one whole system/service. Dashboard aspects that should be evaluated include end user interfaces (ease of use, customisability, feedback to users, human error tolerance, etc.), knowledge discovery, public health information delivery and communication, system security, and integration and interoperability with other relevant/existing public health systems [55].

### Task cluster 7: research and technological development (R&D) coordination and management

Designing effective public health dashboards is a highly multidisciplinary and interdisciplinary undertaking that must address a number of essential criteria and tasks concurrently. These tasks cover (1) research integrity, quality assurance and system/evidence updates, (2) multidisciplinary collaboration, (3) risk management, and (4) exploitation of the demonstrator's results.

Firstly, a dashboard should achieve scientific rigour. Data, analytics methods, visualisation and underlying research design, as well as the underpinning clinical/public health evidence must all be robust and sound, avoiding the well-documented failings and traps of big data [56]. Dashboards must be designed and developed in such a way that accommodates and streamlines future maintenance and expansion updates of software components (functionality, security, etc.), consumed datasets, and underpinning clinical/public health evidence and guidelines (as science progresses). Updates (and the mechanisms for implementing them) ensure the quality of the dashboards can be maintained and improved over time. Standard best practices in research ethics and individual privacy preservation must be adhered to.

Secondly, it is vital to build and maintain strong partnerships, close collaboration and mutual understanding between the multidisciplinary members of the demonstrator team (with their different but complementary professional backgrounds and areas of specialism), target users and other stakeholders. The research literature offers some excellent discussions about participatory practices and platforms in dashboard building that should prove helpful in fostering and facilitating these collaborative partnerships [57, 58].

Thirdly, risks assessment and mitigation plans ('plan B') must be put in place at the very start of the work on the demonstrator. Lastly, outputs dissemination activities and exploitation plans should be developed to keep the wider stakeholders and managers engaged and supportive of the work, secure continuing funding, and ensure future viability and expansions of the demonstrator, both in functionality and to other cities/regions and populations.

### Looking forward to research in 2025 and beyond

Big data approaches in genomics, epigenomics and bioinformatics, coupled with geographic information (systems), are unveiling the complex interplay of environment, genes and other factors in health and disease [59]. Genes confer potential protections and predispositions. But it is the lifestyle/environmental (exposome) modulation – up and down, on and off – of their expression (gene expression) via the epigenome (epigenetics) that determines their ultimate effects, i.e., can increase/enhance or decrease/mute the negative (and positive) effects of lifestyle and environmental exposures on the individual, depending on the unique interplay between a person's genetic profile and her/his lifestyle and environmental exposures [60]. Some of the epigenome-tagged genome DNA and histones can even be heritable. Environmental pollutants (obesogens), gut microbiota modifications and unbalanced food intake can induce, through epigenetic mechanisms, OO and altered metabolic consequences [61].

But that is not the end of the story; we can still 'tame' (tone down/turn off the expression of) unfavourable genes with suitable external factor modifications and interventions, e.g., by introducing targeted gamified exercise interventions (exergames/mobile exergame apps) for added exercise 'appeal' and PA behavioural sustainability in those individuals and population groups that 'hate' exercise because of their genetic makeup [62].

Indeed, future public health interventions will evolve to precisely identify, and address the specific characteristics and needs of, target population groups or areas, inasmuch the same way as personalised precision medicine is evolving in managing individual patients. In the future, we will know more about our target populations, thanks to population genome data banks, where profiles of large local population samples can be mined and analysed for the presence specific relevant genes, gene variants/mutations and dysfunctions, such as those genes controlling appetite, or implicated in overweight and obesity predisposition, or determining our base exercise behaviour, e.g., fat mass and obesity-associated (FTO), melanocortin-4 receptor (MC4R), leptin (LEP), etc. [63]. We will then use this intelligence to inform the design of optimised individual- and population-group-specific interventions that can make our genes work best for us and/or offset their negative effects (predispositions).

Similarly, we can have population gut microbiome data banks of large local population samples (with regularly updated profiles, say every 6 months, to monitor change). A growing number of consumer-oriented labs are already offering 'gut bacteria profiling' services today at affordable prices. Gut bacteria types and diversity/ratios (some gut bacteria are 'bad' in relation to obesity, e.g., *Firmicutes*, while others are 'good', e.g., *Bacteroidetes* [64]) can be modified or modulated/influenced, as necessary, through both individual- and population-group-specific interventions to make our gut microbiomes work best for us, e.g., through targeted interventions involving specific dietary and lifestyle modifications/mass health education about diet, such as encouraging the consumption of high-fibre diet, sauerkraut (cabbage), yoghurt and kimchi, etc., all of which are known to promote good gut bacteria.

### A few additional notes

It should be noted that the demonstrator discussed above is all about making sense of 'crude' population data aggregates for public health purposes. Therefore, it is not about collecting ultra-precise individual patient data or the precise clinical management of individual patients. Moreover, there will be other research opportunities related to this demonstrator that were not fully developed or described in this article. For example, task clusters 4 and 5 can additionally help generate new hypotheses for further clinical and epidemiological research beyond the scope of this demonstrator, as an extra advantage of having such big population lifestyle data at hand. An open platform/API (application programming interface) or a 'data cooperative' can be offered as one of the demonstrator's outputs to enable other research groups, nationally and internationally, to interrogate and interact with select sets of the demonstrator's data and analytics, subject to governing data regulations and adequate data privacy and security safeguards. The companion ‘Additional file 1’ provides further important details and pointers to the research literature that complement the material presented in this article.

## Conclusions

Geo-tagged big data from smartphones, wearables and other sensors are enabling researchers to conduct innovative studies in OO and T2D. (Smartphones are not just useful for data collection; they can also be used to deliver some location-based targeted public health interventions and campaigns.) Public health professionals can greatly benefit from well-conceived big data dashboards and related technologies in unveiling and acting upon the multifaceted challenges of OO and T2D in their target populations. The roadmap presented in this article with its list of high-level task clusters should provide a good start for teams willing to develop these dashboards for smarter public health decision-making in OO and T2D control and prevention in their locales. Dashboards must always be designed and developed in such a way that accommodates and streamlines future updates of not just the software components, but also the consumed datasets (e.g., adding new population genetic/epigenetic datasets and population gut microbiome profiles/trends when they become available) and the underpinning clinical/public health evidence and guidelines (as science progresses). It is hoped this article will initiate and stimulate further fruitful discussions among public health communities worldwide, and inspire many future ground-breaking studies about food and PA environments and population factors in OO and T2D.

## Disclaimer

Reference in the manuscript to any specific commercial product, process or service by trade name, trademark, manufacturer or otherwise does not necessarily constitute or imply its endorsement, recommendation or favouring by the authors or the entities they are affiliated to, and shall not be used for commercial advertising or product endorsement purposes.

## Supplementary Information

**Additional file 1.** A feasibility demonstrator roadmap (supplementary slide set).

## Data Availability

Data sharing is not applicable to this article, as no datasets were generated or analysed for the current paper.
