# Supplementary material for: Smart city lifestyle sensing, big data, geo-analytics and intelligence for smarter public health decision-making in overweight, obesity and type 2 diabetes prevention: the research we should be doing
Source: Int J Health Geogr. 2021 Mar 3;20:12. doi: 10.1186/s12942-021-00266-0 (PMC7926080; doi:10.1186/s12942-021-00266-0)
Supplement: Supplementary file 1 — Additional file 1. A feasibility demonstrator roadmap (supplementary slide set). [file 12942_2021_266_MOESM1_ESM.pdf]

Smart city lifestyle sensing, geo-analytics and intelligence for smarter public health decision-making

Towards precision public health interventions for overweight, obesity (OO) and type 2 diabetes (T2D) prevention

A feasibility demonstrator roadmap

MN Kamel Boulos (mnkboulos@ieee.org; mnkboulos@mail.sysu.edu.cn)

About this slide set: (Version date: 29-12-2020)

- OO and T2D are super multifactorial and deeply interrelated = a '**systems thinking**' approach is a must
- The main focus of this slide set is on *what lifestyle and environmental data **can be relevant** in these conditions*, but ***not*** on what data are readily available, or feasible to collect, in an appropriate form in Guangzhou/China, which is used throughout this slide set as an internationally representative case/locale!

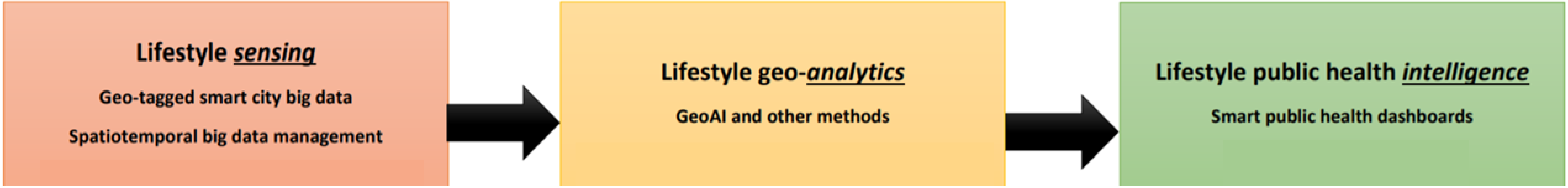

Our ultimate goal: precision public health

- Term first introduced by Khoury et al. in 2016: **Precision Public Health for the Era of Precision Medicine** https://www.ncbi.nlm.nih.gov/pmc/articles/PMC4915347/
- A misnomer; should have been called **Accuracy Public Health**, but precision public health sounds nicer. Actually, we need both in public health: accuracy and precision

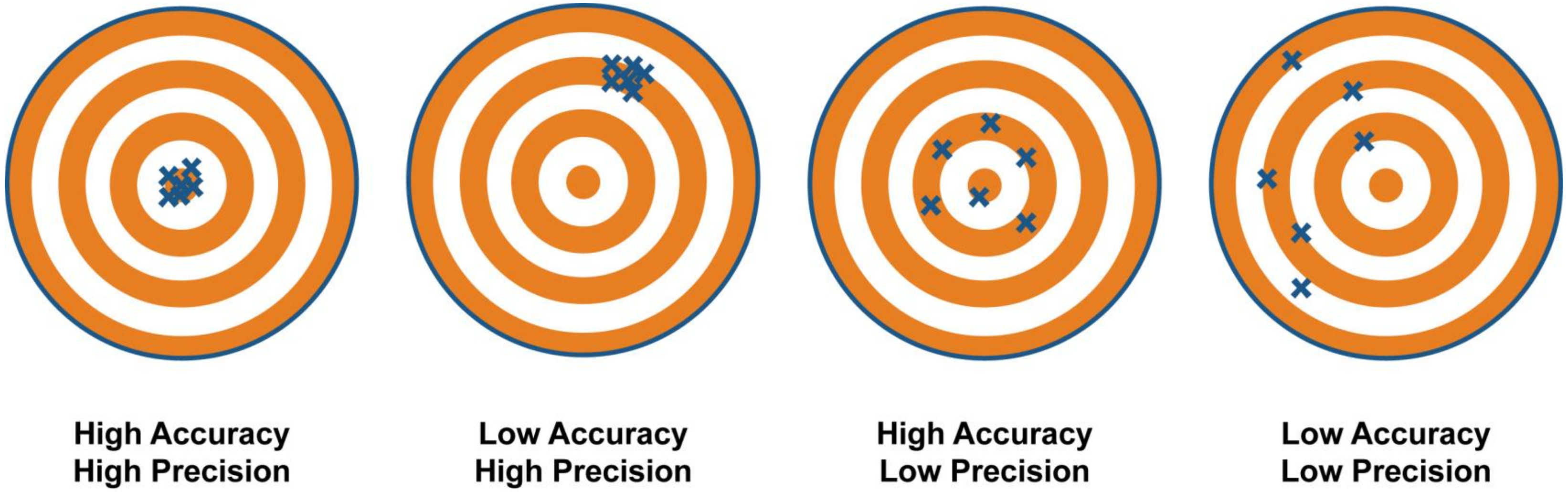

Clipart via
https://binged.it/2MeWyTB

Challenges in precision public health

- Quoting Khoury et al., 2016: “***Separating signal from noise*** *will not be easy. A healthy dose of scepticism may be needed to guard against the* ***overpromise of big data****. For example, in 2013, when influenza hit the U.S. hard, Google monitored the outbreak using analysis of influenza-related Internet searches, drastically overestimating peak influenza levels, compared with public health surveillances. Even more problematic could be the potential for many* ***false alarms*** *by mindless examination, on a large scale, leading to putative associations between big data points and disease outcomes. This process may* ***falsely infer causality and could potentially lead to faulty interventions****. Separating signal from noise will require epidemiologic study designs that minimise bias, robust knowledge integration process, adherence to principles of evidence-based medicine and population screening, and a robust multidisciplinary translational research agenda that goes beyond initial discoveries to implement findings in populations.*”
- See Lazer et al., 2014: https://science.sciencemag.org/content/343/6176/1203.full

The Parable of Google Flu: Traps in Big Data Analysis

David Lazer[1,2,*], Ryan Kennedy[1,3,4], Gary King[3], Alessandro Vespignani[5,6,3]
+ See all authors and affiliations

Science 14 Mar 2014:
Vol. 343, Issue 6176, pp. 1203-1205
DOI: 10.1126/science.1248506

Overweight and obesity in China

- **Overweight:***
  - **China** has the largest overweight population in the world, bumping the United States to second place
- **Obesity:****
  - **China** has the highest numbers of obese **children** in the world
  - The United States followed by **China** have the highest numbers of obese **adults** in the world
- **Overweight and obesity are a documented leading risk factor of major non-communicable diseases**, including **type 2 diabetes (and its many complications), heart disease, joint disease, and certain types of cancer and dementia**

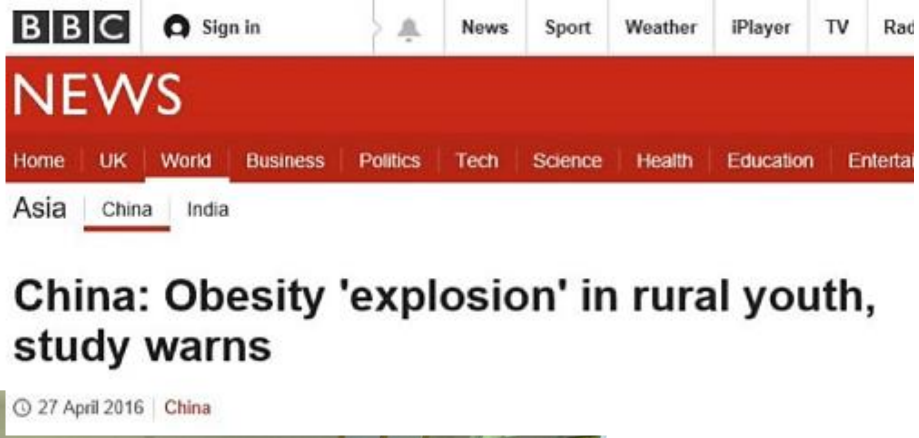

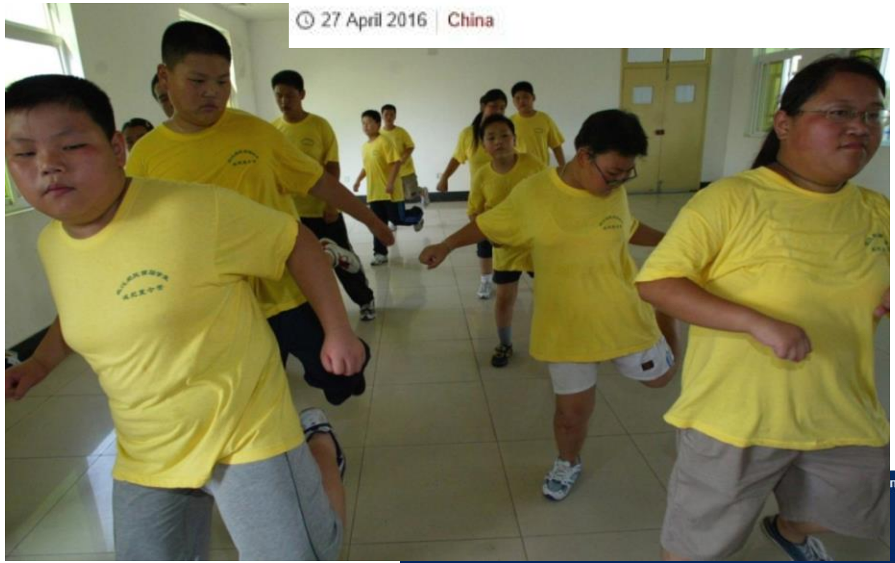

< Summer camp for overweight youngsters in Wuhan (AP – fair use)

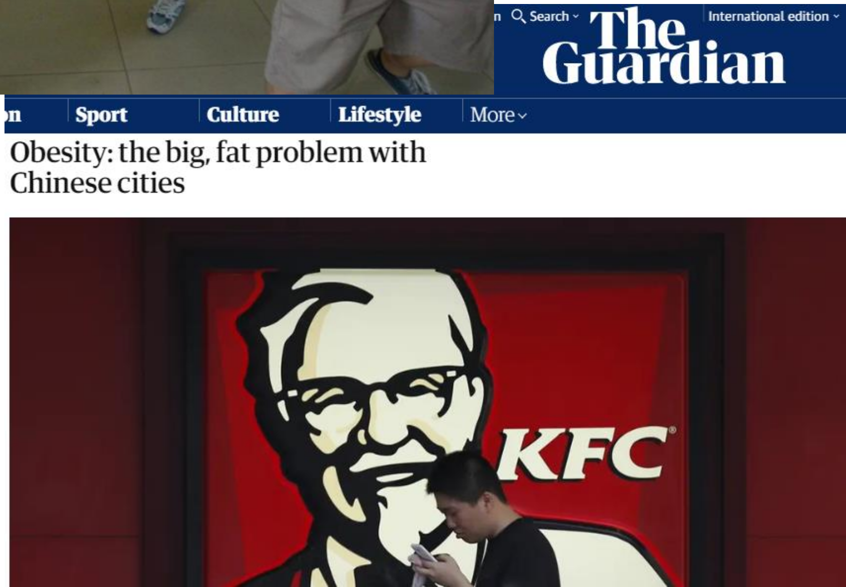

Additional China-specific studies are presented on next slide

* Trends in adult body-mass index in 200 countries from 1975 to 2014
https://www.thelancet.com/journals/lancet/article/PIIS0140-6736(16)30054-X/fulltext

** Health Effects of Overweight and Obesity in 195 Countries over 25 Years
https://www.nejm.org/doi/full/10.1056/NEJMoa1614362

Overweight and obesity in China

Use to extract China-specific factors contributing to OO, incl. demographics*

- Liu et al., 2018: Prevalence and influencing factors of overweight and obesity in a Chinese rural population: the Henan Rural Cohort Study https://www.ncbi.nlm.nih.gov/pmc/articles/PMC6117281/
- He et al., 2017: Prevalence of overweight and obesity in 15.8 million men aged 15–49 years in rural China from 2010 to 2014 https://www.nature.com/articles/s41598-017-04135-4
- Hu et al., 2017: Prevalence of overweight, obesity, abdominal obesity and obesity-related risk factors in southern China https://journals.plos.org/plosone/article?id=10.1371/journal.pone.0183934
- Zhou et al., 2017: Prevalence of overweight and obesity in Chinese children and adolescents from 2015 https://www.tandfonline.com/doi/abs/10.1080/03014460.2017.1371224
- Wang et al., 2016: Prevalence of overweight and obesity and some associated factors among adult residents of northeast China: a cross-sectional study https://bmjopen.bmj.com/content/6/7/e010828
- Xu et al., 2016: Prevalence of Overweight and Obesity among Chinese Adults: Role of Adiposity Indicators and Age https://www.karger.com/Article/FullText/443003
- **More China-specific studies:** https://www.ncbi.nlm.nih.gov/pubmed/?term=(overweight+OR+obesity)+AND+China

* Evidence-based public health: locale- and population-specific evidence will be vital for the success of this approach

Obesity drives the high prevalence of type 2 diabetes in China (and elsewhere in the world); the two conditions are syndemic

- **China now has the world's largest and fastest growing diabetes epidemic**: recent prevalence figures in China are ~11% for diabetes and ~36% for prediabetes*
- Up to 10% of people with prediabetes will progress to diabetes each year**
- **Asians** are at increased diabetes risk at lower body weights (i.e., at lower levels of overweight and obesity, about 7 Kg lower) than people from other parts of the world, as they tend to accumulate more visceral fat within the same BMI range^ compared with Westerners***
- The resulting **burden** of disease, death and disability and the costs of managing it are huge, **with significant negative economic and productivity impacts**

^ TOFI = Thin Outside Fat Inside

+ The cost of managing diabetes and its complications in China alone is: >¥1.1 trillion CNY per year*

+ It is possible to prevent the progression to diabetes in people with prediabetes

* http://dx.doi.org/10.1016/S0140-6736(16)00618-8

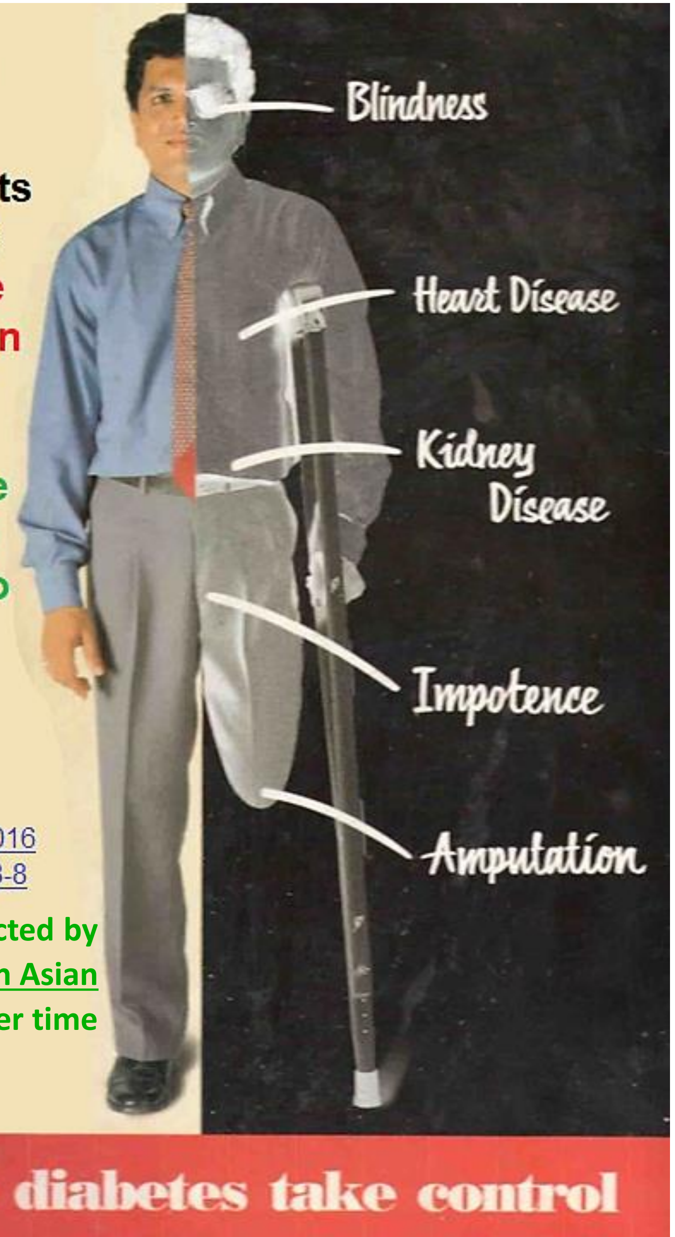

+ Preventing disease progression and complications: The majority of patients affected by obesity and type 2 diabetes are young or in their early middle age (particularly in Asian populations) and will accumulate and have greater exposure to disease complications over time

* Wang L, Gao P, Zhang M, et al. Prevalence and Ethnic Pattern of Diabetes and Prediabetes in China in 2013. JAMA. 2017;317(24):2515-2523. doi:10.1001/jama.2017.7596
** https://www.ncbi.nlm.nih.gov/pmc/articles/PMC3891203/
*** https://www.ncbi.nlm.nih.gov/pmc/articles/PMC4595349/

Smart city lifestyle sensing for well-targeted public health interventions – Process flow diagram

© 2019-2021 MN Kamel Boulos

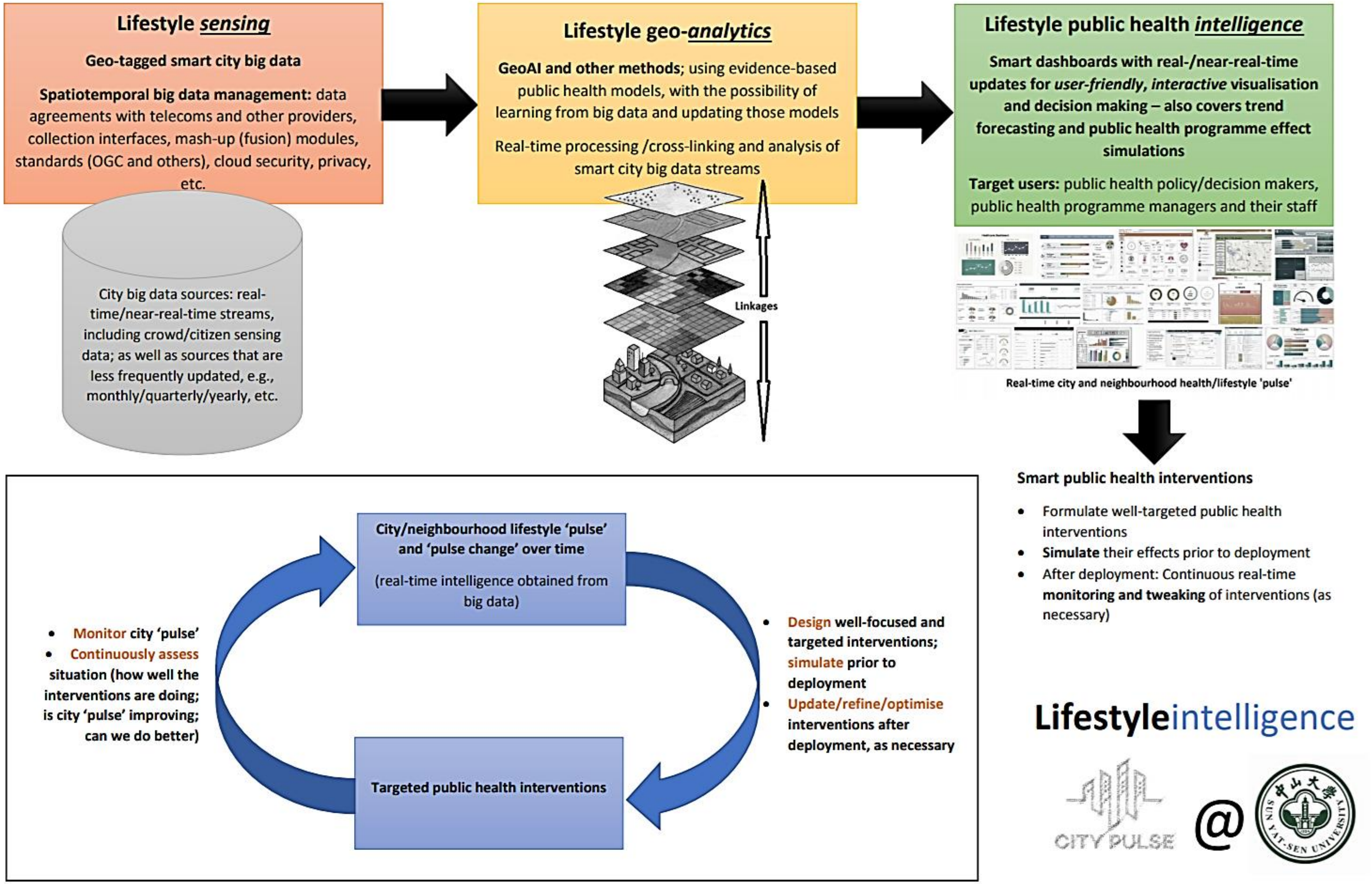

- Our representative case study city could be Guangzhou (population >13M), with the option to include an additional city (in China or elsewhere), if possible and if the corresponding data/access are available to us. We can then produce some interesting comparisons between the two cities once our demonstrator is up and running*

Determine, and regularly update details of, the most problematic **neighbourhoods**, population **groups** in most need, and ***where*** and ***how*** to best **target** them with appropriate public health interventions/campaigns

Geo-tagged data are essential to realise this vision!

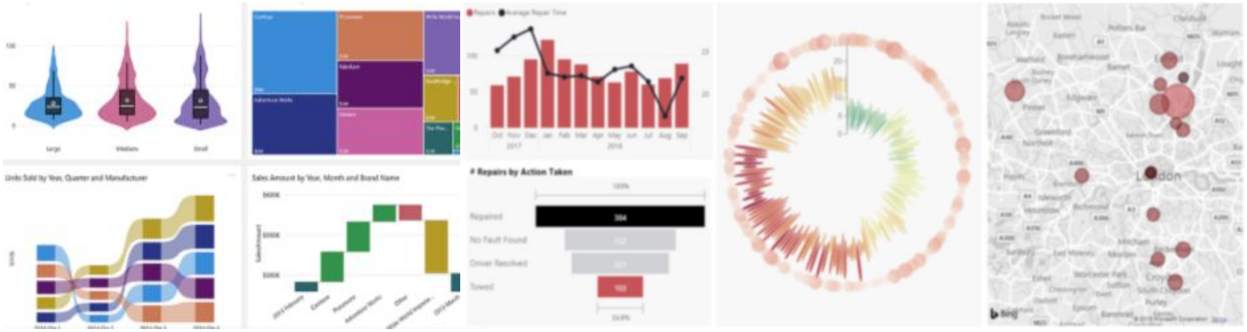

^ Generic MS Power BI clipart

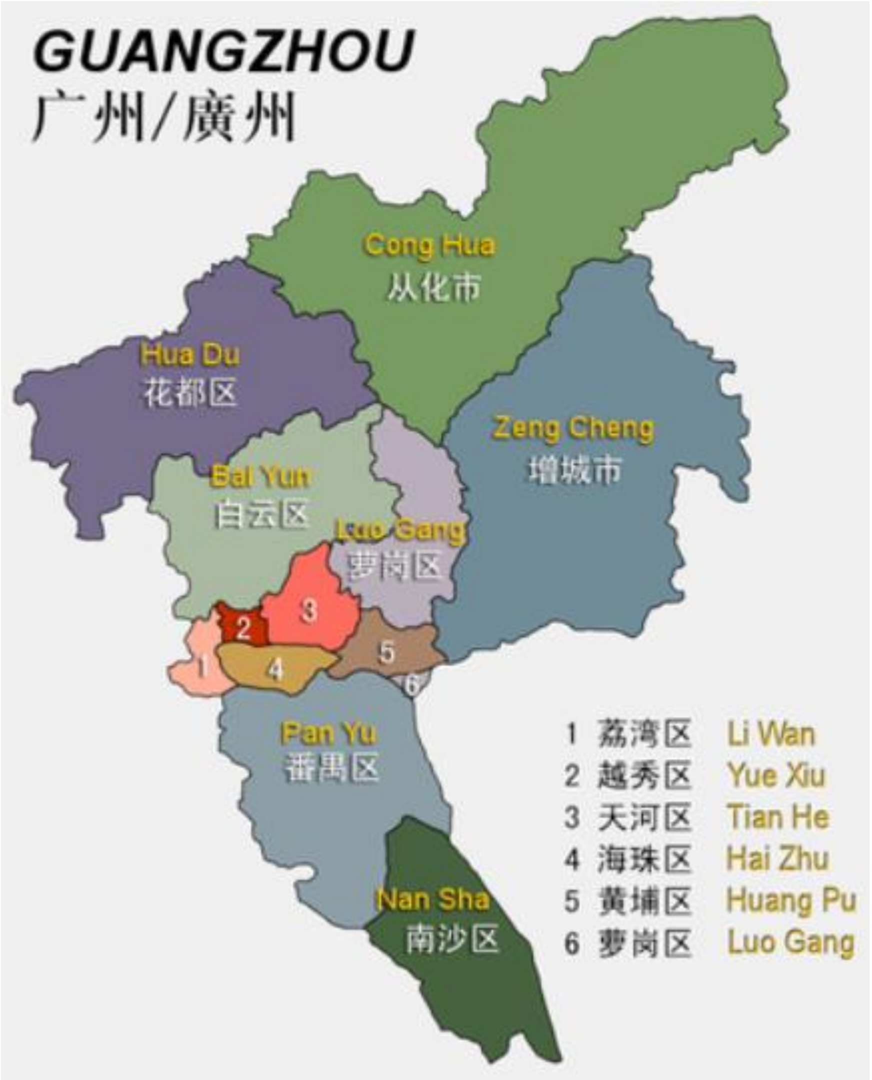

* Expected Technology Readiness Level at the end of demonstrator development and evaluation is TRL6 or TRL7

Demonstrator development and evaluation

Task clusters

*** Task cluster 1: Agree a comprehensive list of key population lifestyle data that have a high correlation to the prevalence of OO and T2D**

-- *Later in this slide set, I will provide some examples of relevant population lifestyle data can be collected (just to help kick-start this task; this is not meant to be an exhaustive list of all items one should/can consider)*

*** Task cluster 2: Identify the most efficient and appropriate/reliable sources, modes and frequency for collecting different lifestyle data**

--e.g., in real-time vs. every two or three months, depending on nature/source of data and their mode of collection. Later in this slide set, I will introduce some of the challenges faced when using wearables

*** Task cluster 3: Big data issues**

--Big data governance and management, data and metadata standards (esp. OGC http://www.opengeospatial.org/standards*), data quality, ontologies and data warehousing, secure cloud storage, compliance with data protection regulations, individual data privacy issues if non-aggregate data are collected, data sharing agreements with providers, etc.*

*** Task cluster 4: Data fusion and geo-analytics (GeoAI and other methods)**

--Involves data integration, including the challenges of managing data uncertainties and incompleteness/missing data points across fused data-sets, etc. Also involves developing own custom analytics tools and solutions using own (difficult) or existing (easier) platforms, such as MS Power BI https://powerbi.microsoft.com/ and its ecosystem of software extensions, e.g., https://dataveld.com/2017/06/03/10-ways-to-create-maps-in-microsoft-power-bi/

Power BI

*** Task cluster 5: Smart public health dashboards, including mechanisms for intervention simulations and near-real-time monitoring and optimisation of interventions**

--Intelligent interactive visualisations of the generated intelligence; intelligent alerts and reminders inferred from the combined patterns of change of multiple interrelated parameters (beyond mere threshold-based triggers derived from single data sources); user-friendly interrogation of the data in different ways

*** Task cluster 6: Multi-faceted evaluation (usability, utility, accuracy/reliability, etc.) of the interfaces and tools created in Tasks 4 and 5 above**

*** Task cluster 7: R&D coordination and management, including research ethics, risks assessment and mitigation, as well as outputs dissemination and exploitation activities**

User requirements analysis (should form part of Tasks 1, 2, 4 and 5)

- **Public health professionals (the users of the planned dashboards)** requirements analysis: what are their information/intelligence needs in relation to OO and T2D prevention
- We need to investigate and learn about their current (conventional workflows): their current public health interventions in OO and T2D and the sort of information that feeds into the design of those interventions and is used in (manually) monitoring their implementation/success; what kind of information will help them do a better task and improve their workflows, ultimately resulting in better public health outcomes with reduced costs; etc.
- Ensure continuous user involvement throughout the whole demonstrator development effort from start to end, and not just during the user requirement analysis and evaluation stages
- Develop iteratively, and regularly seek and incorporate users’ feedback as the work progresses
- Fully understanding our users’ needs will be key to the successful prioritisation of demonstrator choices and developments/phases in a climate of finite (limited) resources and budgets

Important notes

- This demonstrator is all about making sense of **crude** population data **aggregates** for **public health** purposes (*cf.* syndromic surveillance https://www.esri.com/news/arcuser/0206/geostat1of2.html); it is not about collecting ultra-precise individual patient data or the precise clinical management of individual patients
- As a bonus (and to help us further 'sell' our ideas to our funders and reviewers), Tasks 4 and 5 above can also help **generate new hypotheses** for further clinical and epidemiological research (**beyond the scope of this demonstrator**), as an extra advantage of having such big population lifestyle data at hand. As one of our demonstrator outputs, we can offer an open **platform/API or a 'data cooperative'** for other clinical research groups (nationally and even internationally) to interrogate/interact with our data and analytics, subject to existing/governing data regulations and safeguards

See Park et al., 2018: https://www.ncbi.nlm.nih.gov/pmc/articles/PMC6230537/

Digital Epidemiology: Use of Digital Data Collected for Non-epidemiological Purposes in Epidemiological Studies

Hyeoun-Ae Park, PhD, FAAN, FACMI, RN, Hyesil Jung, MSN, RN,[⊠] Jeongah On, RN, Seul Ki Park, RN, and Hannah Kang, MSN, RN

Lifestyle data and data sources (Tasks 1 and 2)

- **Evidence-based selection:** The complex interplay between lifestyle and OO/T2D has already been very well researched and documented in the peer reviewed literature, so we should start from there, reviewing the latest literature on the subject. In fact, we do have lifestyle medicine scholarly journals for some years now, in addition to traditional journals specialising in diabetes, obesity, public health, etc., which also regularly publish on this topic

- For example: Kolb and Martin, 2017: **Environmental/lifestyle factors in the pathogenesis and prevention of type 2 diabetes**
  https://www.ncbi.nlm.nih.gov/pmc/articles/PMC5516328/

  Factors (= data classes/topics): diet quality and quantity, smoking, little physical activity, increased screen viewing time or sitting in general, exposure to residential traffic, noise, and fine airborne particulate matter (fine dust), short or disturbed sleep, smoking, stress and depression, and a low socioeconomic status

- Interesting fact: A non-diabetic partner of a diabetic spouse has a 26% increased diabetes risk, likely due to a **'contagious' lifestyle**
  https://www.ncbi.nlm.nih.gov/pmc/articles/PMC3900990/

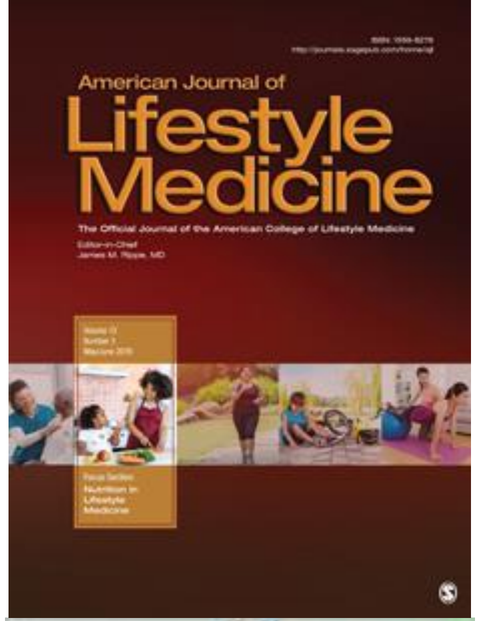

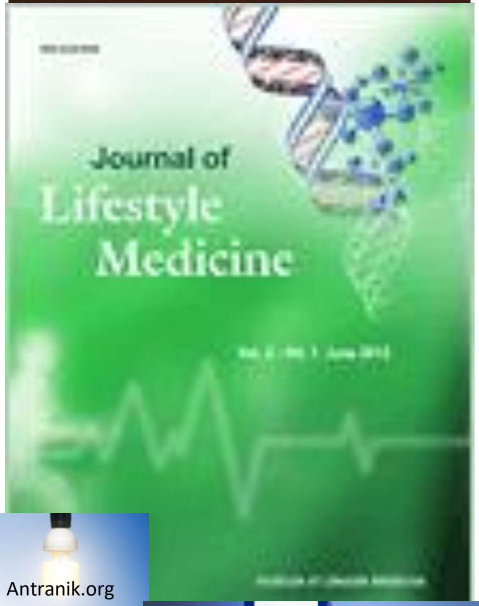

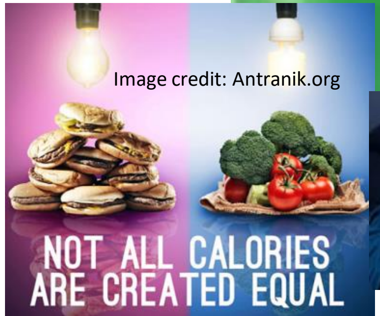

Image credit: Antranik.org

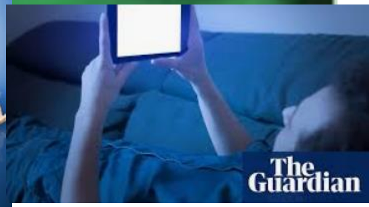

^ Screen time - Image credit: the Guardian

Box 1 (right) source: Kolb and Martin, 2017 (see previous slide)

Sitting ~= smoking

Below: Side topic, unrelated to the paper by Kolb and Martin

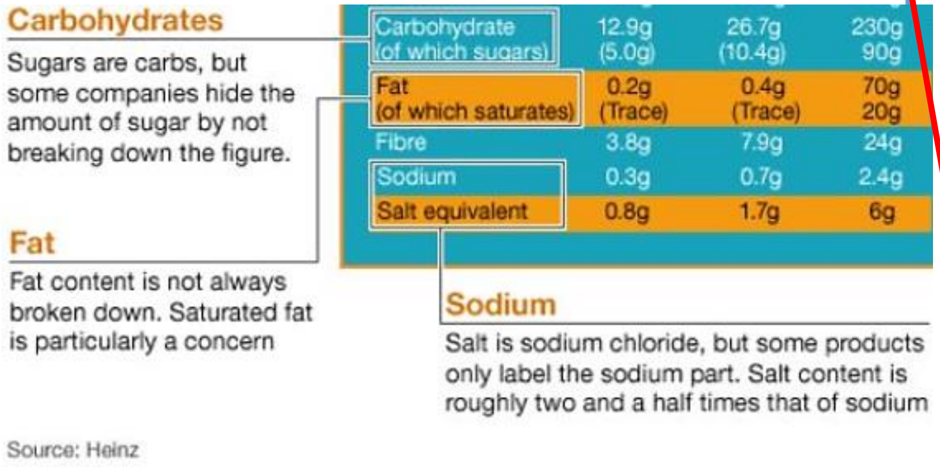

Vital information is missing, leaving consumers disadvantaged: Unlike in the UK and the EU, Chinese-language nutrition facts labels on most (I would say >90% of) foods and drinks produced in China, as sold at supermarkets and other retailers in Guangzhou, only show the total carbohydrates and total fat figures (e.g., in g/100g of the product) but hide the essential breakdown figures of sugars and saturated fat. (This is not the only problem with these nutrition facts labels)

BMC Med

Box 1. Lifestyle characteristics conferring risk[a] for type 2 diabetes as suggested by epidemiological studies

Go to:

Diets poor in fibre, phytochemicals or plant food in general (relative risk increase by 44% to three-fold [14, 15])

Regular consumption of sugar-sweetened beverages (relative risk increase by 20–30% compared to non-consumption [22–24])

Little physical activity (leisure time/occupational) (relative risk approximately 40% higher compared to high total physical activity [41, 42])

Prolonged TV and monitor viewing/sedentary time (relative risk increased by approximately 3% per hour television watching [49])

Exposure to road traffic (noise, fine particulate matter) (relative risk increased by 20–40% for exposure to 10 dB higher noise level or 10 μg/m^3 more of fine dust particles [55–59])

Smoking (relative risk increased by approximately 30%/60% for light/heavy smokers [85])

Short sleep duration and poor quality (relative risk increased by approximately 9% for every hour of shorter sleep duration [63])

Low mood/stress/depression (relative risk increase highly variable, depending on definition of stress and depression)

Low socioeconomic position (relative risk increased by 40–100% when compared to high socioeconomic level [99–101])

Infection with hepatitis C virus or *Chlamydia pneumoniae* (no epidemiological data on relative risk increase available)

[a]All factors remain significantly associated after statistical adjustment for body mass index (BMI) and other confounders, as described in the text. Virtually all non-infectious pro-diabetic lifestyle characteristics promote an increase in BMI and waist circumference.

Environmental factors, such as the various types of environmental pollution, as well as **socioeconomic and occupational** factors, including household income, **influence and are influenced by** population **lifestyles** through a wide range of different mechanisms and pathways (Kamel Boulos et al., 2021)

Activity spaces (present and past): Humans are not confined to a single address point/postcode all the time/all their life, and diseases are often the product of both present and past exposures. "If time spent walking outdoors and biking is relevant for the exposure to environmental factors, then relying on (just) the home (or work/school) address as a proxy for exposure location may introduce misclassification" (Klous et al., 2017)

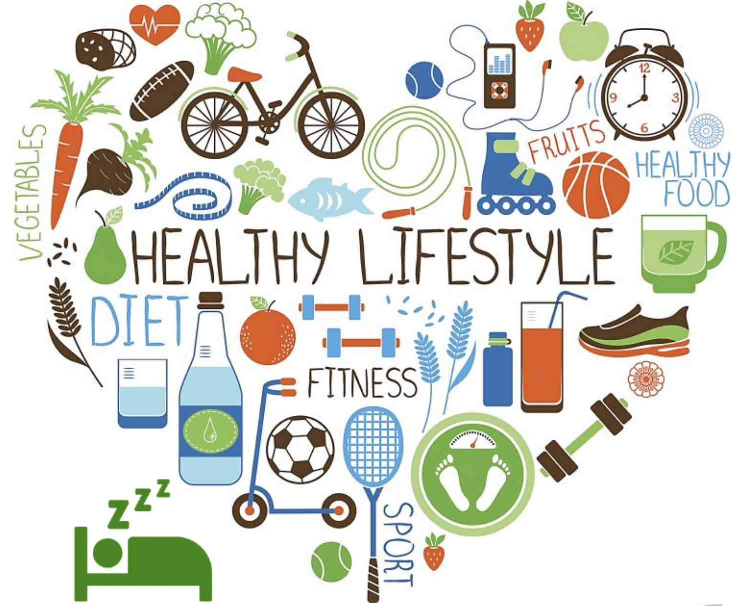

Stock clipart credit: 123RF and others

Some clipart depictions on the right are not scientifically accurate. For example, recent peer reviewed studies have shown that a large glass of 100% pure no-added-sugar fruit juice a day increases risk of premature death, and that excessive/extreme exercises can lead to irreparable cardiovascular damage and premature death (even in young people)! '*In moderation*' is the key phrase here!

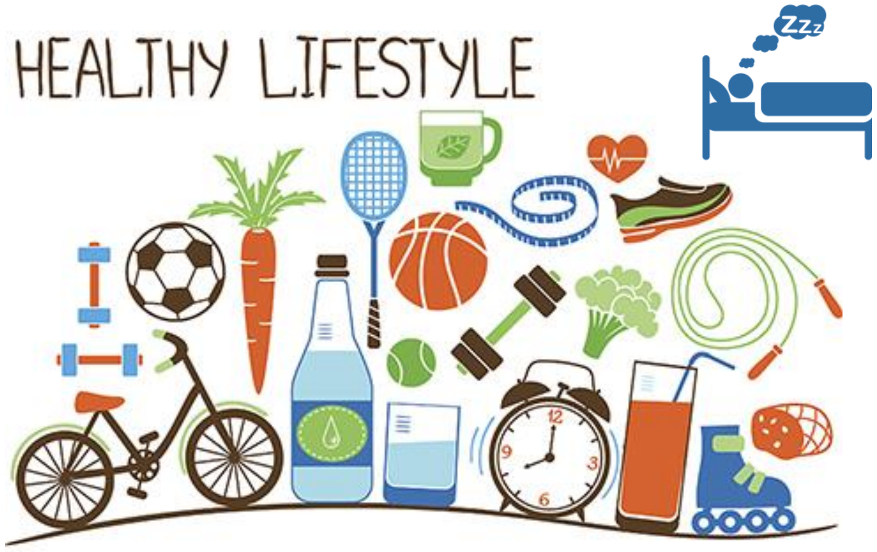

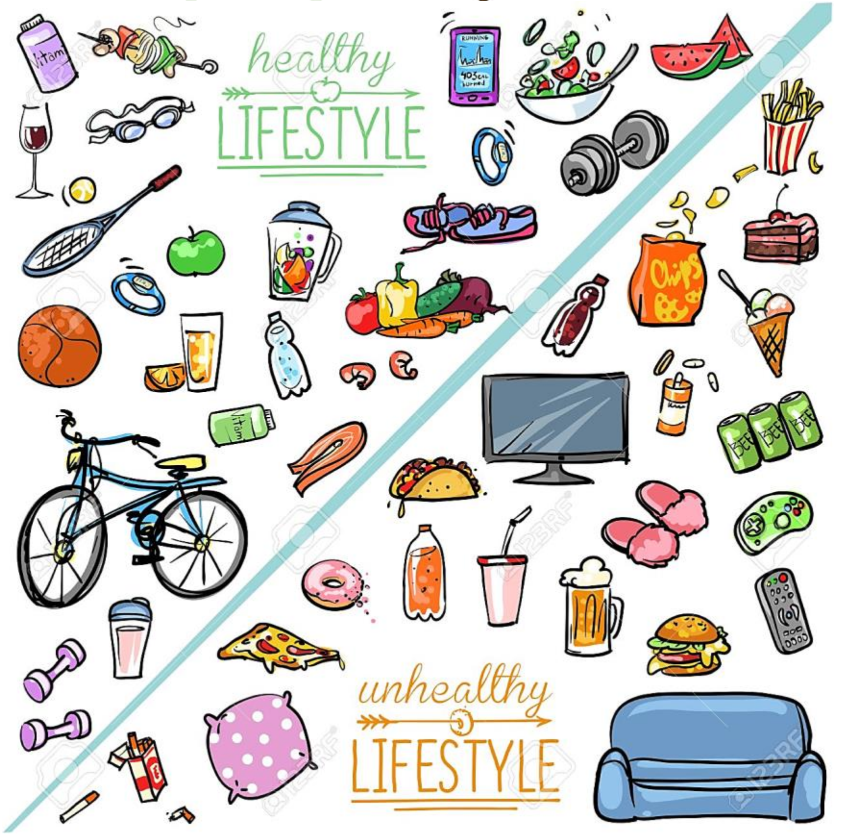

Lifestyle data and data sources (Tasks 1 and 2)

- Some of the factors/items on the previous slide are easy to collect from large populations of infer from existing big data that are routinely collected for other purposes (e.g., semi-annual clinical data aggregates from citywide hospitals/clinics, census data [every 10 years], etc.), with minimal or no individual subject inconvenience; some are more difficult/costly to collect or require surveys of representative samples of the population or other types of population sampling
- **Mobile crowdsensing:** mobile data and telemetry collected without any, or with minimal, user intervention, e.g., obtained from general smartphone apps already in wide use or from purpose-built apps (with or without sensors such as a fitness/sleep tracking band) introduced in a suitable public health campaign,* with appropriate incentives, such as free mobile minutes or data, free tracking band, etc., to encourage large numbers of people to download them (those same purpose-built apps can also serve as a vehicle for serving tailored public health interventions to the public)
- **Data sampling rates:** in real-time, or near-real-time, or non-real-time (e.g., updated monthly or 2-4 times every year or yearly). Some data are not available in real-time, while other data, though available in real-time, will not be needed, for our purposes, at that frequency and can be reasoned with in monthly or less frequent aggregates

* e.g., Noise pollution monitoring by citizens (app) http://noisetube.net/ and air quality monitoring by citizens (bike sensors + app) http://cambikesensor.net/ and the Air Quality Egg (IoT device for crowdsourced citizen monitoring of airborne pollutants) https://airqualityegg.com/

* Example of a public diabetes portal introduced in 2019 by England's NHS that will not only benefit individual patients, but also accumulate big health data that can inform future population interventions https://www.dailymail.co.uk/health/article-7076217/Diabetes-patients-encouraged-care-health-using-personalised-website.html

Lifestyle data and data sources (Tasks 1 and 2)

- City food/diet patterns: **China Food Composition Database** (Institute of Nutrition and Food Safety, China CDC, Beijing, 2002, 393 pp. [Chinese & English]) http://www.fao.org/infoods/infoods/tables-and-databases/china/en/ and other food databases applicable to China can be used to identify relevant classes/types of food and **segment** city food outlets and city food retailers' sales figures accordingly
- Neighbourhood food POI segmentation, such as outlets serving ultra-processed food*, high GI (glycaemic index) food and food high in High Fructose Corn Syrup—HFCS vs. those selling unprocessed or minimally processed foods and low GI food; also alcohol and tobacco outlets, red meat, vegetables/fruits and soda/sugary drinks/fruit juice outlets data are all important; etc.
- Data sources: **Population surveys** (food types and quantities, tobacco, smoking, etc.); city **segmented consumption levels** (incl. avg. per capita); **city segmented food and drink sales** (e.g., major supermarket chain(s), food factories, fast food chains, etc.); alcohol and tobacco sales (e.g., annual sales of pure alcohol in litres per person aged 15 years and older) and related aggregate data derived from hospital records; meat and processed meat consumption/sales/outlets, esp. red meat (high consumption of red meat/animal saturated fats = increased risk of T2D); vegetables and fruit/plant-based food consumption levels/sales/outlets; drinks containing HFCS, soda (even 'diet' varieties with artificial sweeteners), other sugary drinks and fruit juice consumption levels/sales/outlets; etc.

- Hall et al., 2019: Ultra-Processed Diets Cause Excess Calorie Intake and Weight Gain https://www.cell.com/cellmetabolism/fulltext/S1550-4131(19)30248-7
- Collin et al., 2019: Association of Sugary Beverage Consumption With Mortality Risk in US Adults https://jamanetwork.com/journals/jamanetworkopen/fullarticle/2733424 (unlike whole fruits, fruit juice, even the varieties that claim to be pure or with 'no added sugar', are not that healthy!)
- Aprelini et al., 2019: High consumption of red meat increased by 40% the likelihood of new cases of diabetes in Brazilian men https://www.ncbi.nlm.nih.gov/pubmed/31093264
- Goran et al., 2013: Countries with higher availability of HFCS have a higher prevalence of type 2 diabetes independent of obesity https://www.ncbi.nlm.nih.gov/pubmed/23181629
- Basciano et al., 2005: Fructose, insulin resistance, and metabolic dyslipidemia https://www.ncbi.nlm.nih.gov/pmc/articles/PMC552336/
- Diet/artificially-sweetened beverages are associated with increased obesity and T2D risks; see, for example: https://academic.oup.com/ajcn/article/97/3/517/4571511 and https://onlinelibrary.wiley.com/doi/abs/10.1111/jgs.13376

Food type segmentation – Not all calories are equal

- An important research gap in the current research literature is the segmentation of food consumption in large populations (per capita) by food type, and the associations of this segmentation/segmentation patterns with different population characteristics and of course overweight and obesity
- It is the type (and relative quantities) of diet that matters the most in overweight and obesity, and not just the total number of calories consumed, the amount of physical activity undertaken, or the proximity to supermarkets or KFCs. The same food store, e.g., supermarket, KFC or other fast food outlet/takeaway, can often sell/serve good and bad calories, healthy and non-healthy options/portions. 100 calories from a 250ml sugary beverage and 100 calories from a handful of nuts are not the same. The segmentation of calories is essential, as not all calories have equal effect with respect to obesity, or as they say ‘a calorie is not a calorie’
- The type of food outlet/food type sales proportions per outlet, e.g., outlets selling (and proportion of sales of) unprocessed vs. highly processed food; the specific types of food bought by different households as determined by their socioeconomic levels (supermarket loyalty cards/apps are one possible data source here); etc., are the factors that really matter, and the sort of detail resolution we should be investigating and incorporating in our dashboards
- <u>N.B.</u> Individual (and population groups’) dietary/consumption behaviours (amounts consumed per individual snack/meal/day or per family) are also important, as even the healthiest options can prove unhealthy when overconsumed

- Data about socio-economic/demographic profiles, e.g., household income and neighbourhood affluence ('social class' in Western societies) should be factored in, as they often dictate poor diet composition and poor nutrition/food consumption behaviours, both of which are the core issue in overweight and obesity

  Both low cost food and high cost food types/quantities that are typically associated with different socioeconomic levels can result in overweight and obesity and can act as a risk factor for T2D = both poorer and richer neighbourhoods are at risk, but in different ways

- Geo-tagged aggregates of social media streams can be mined and analysed for content,* including images, that promote unhealthy lifestyles/eating or smoking** vs. healthy lifestyles, as well as for the prevailing mood of populations in various parts of a country or region. This knowledge gained can then be used to inform the design of appropriate/targeted public health interventions, including campaigns delivered on social media,*** which can reach out 'virally' to large numbers of people at minimal costs

* e.g., Kamel Boulos et al., 2010: Social Web mining and exploitation for serious applications: Technosocial Predictive Analytics and related technologies for public health, environmental and national security surveillance https://linkinghub.elsevier.com/retrieve/pii/S0169-2607(10)00038-6
** e.g., see some papers at https://www.ncbi.nlm.nih.gov/pubmed/?term=**instagram+smoking**
*** e.g., Kinard, 2016: Insta-Grams: The Effect of Consumer Weight on Reactions to Healthy Food Posts
https://www.liebertpub.com/doi/full/10.1089/cyber.2016.0085

Social media use also contributes a large % of total screen/sitting time https://doi.org/10.1071/HE16026

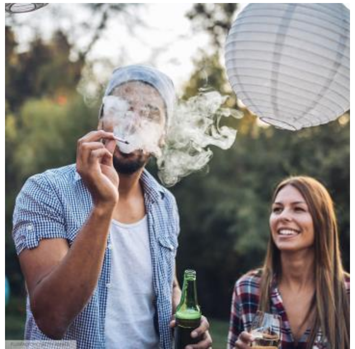

How Big Tobacco Is Using Instagram to Hook a New Generation of Smokers

Tobacco companies are also holding extravagant events with names like "K_Player" and "RedMoveNow" designed to connect with young people.

MAR 27, 2019

https://www.menshealth.com/health/a26965393/big-tobacco-social-media-advertising-cigarettes/

Lifestyle data and data sources (Tasks 1 and 2)

- Physical activity POIs (points of interest) and neighbourhood walkability indicators

  Different factors (= additional relevant data sources to consider) affect neighbourhood walkability and the physical activity levels of its residents, including pollution (air, light and sound [noise] pollution), crowdedness, crime rates, traffic/road network safety, availability and positioning of green spaces, covered sports spaces and gyms (esp. important during temperature extremes and heavy rain/snow seasons), age and gender differences, etc.
  N.B. Road traffic, noise, and fine airborne particulate matter (fine dust) are also risk factors in T2D independent of neighbourhood walkability/physical activity levels

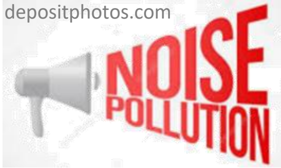

  Also data aggregates obtained by special agreements with mobile telecoms (population daily trajectories/mobility data), app developers, e.g., WeChat WeRun applet (https://blog.wechat.com/tag/werun/), Mi Fit and similar cloud-connected fitness apps that use smartphone GPS and accelerometer data, and which very many people are already using (geo-tagged big data aggregates about population physical activity levels and patterns)

- Population steps data (averages per person/per day) should be considered adequate (cut-off point) at ~7,000 steps/day or 150 minutes of moderate exercise/week (UK Guidance) rather than the popular figure of 10,000 steps/day, which has limited scientific basis*

  * e.g., Lee et al., 2019: "*Among older women, as few as approximately 4400 steps/d was significantly related to lower mortality rates compared with approximately 2700 steps/d. With more steps per day, mortality rates progressively decreased before leveling at approximately 7500 steps/d.*" https://jamanetwork.com/journals/jamainternalmedicine/fullarticle/2734709

  Watch your step: why the 10,000 daily goal is built on bad science

  "There's no health guidance that exists to back the figure of 10,000 steps/day"
  --Mike Brannan, national lead for physical activity at Public Health England

- **Note: Physical activity is undoubtedly critical for good health and disease prevention, including T2D prevention**, but is **not** that good for calorie burning/offsetting excessive calories in diet (our bodies are very energy-efficient machines)! Time to lay to rest that the over-simplistic and inherently flawed 'calories in/out imbalance' model
  Unlearning bad eating behaviours/habits and avoiding HFCS and ultra-processed foods are a healthier, more effective and sustainable long-term strategy than just attempting to burn a couple hundred more calories through exercise

Notes

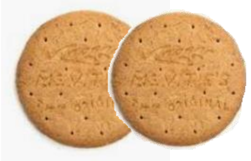

It takes half hour of walking to just burn 2 McVitie's digestive biscuits!

- When it comes to physical activity (PA) tracking apps on smartphones or dedicated wearables/bands (steps/distance, etc.), there is a growing body of research evidence and other reports in the grey literature about the accuracy/inaccuracies of their measurements, which can vary by as much as 50% or more between different models within the same brand/manufacturer and across different brands/manufacturers, **e.g., the distance recorded by a Huawei Watch 2 Sport is typically half that reported by a Samsung Gear S2 for the same individual and activity session!** Also, automatic exercise type detection/segmentation (e.g., walking [slow, brisk, running, stairs climbing?] vs. cycling [road slope?] vs. swimming vs. playing golf or other types of sports, etc., remains under-developed in the current generation of trackers (relying on user's self-reporting is equally inaccurate)
- These inaccuracies would be more relevant and pose potential problems when using the measurements for individual-level interventions, but are **less important in population-level interventions (as in this demonstrator)**, where all what we need are general population PA levels and population trends/changes over time from crude aggregate data from large population samples (devices overestimating PA and those underestimating it are likely to offset one another to some degree)

  Aim for not less than **7000** steps/day (a reasonable cut-off figure for our population analyses). Depending on age/gender and other factors, very little might be gained above that figure; see, for example, [Lee et al., 2019]()

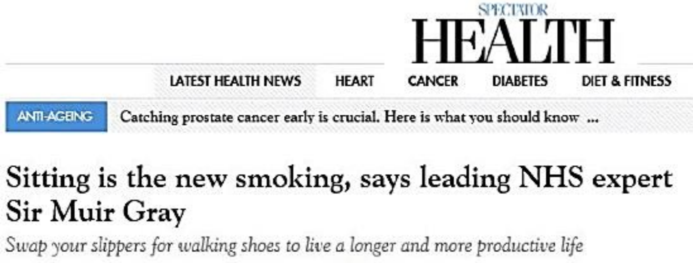

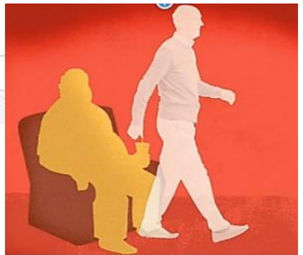

- Malhotra et al., 2015: **It is time to bust the myth of physical inactivity and obesity: you cannot outrun a bad diet** https://bjsm.bmj.com/content/49/15/967.full
  "Coca Cola, who spent $3.3 billion on advertising in 2013, pushes a message that 'all calories count'; they associate their products with sport, suggesting it is ok to consume their drinks as long as you exercise. However science tells us this is misleading and wrong. It is where the calories come from that is crucial. Sugar calories promote fat storage and hunger. Fat calories induce fullness or 'satiation'. A large econometric analysis of worldwide sugar availability, revealed that for every excess 150 calories of sugar, there was an 11-fold increase in the prevalence of type 2 diabetes, in comparison to an identical 150 calories obtained from fat or protein. And this was independent of the person's weight and physical activity level; this study fulfils the Bradford Hill Criteria for causation"
- Brownell and Warner, 2009: **The Perils of Ignoring History: Big Tobacco Played Dirty and Millions Died. How Similar Is Big Food?** https://onlinelibrary.wiley.com/doi/abs/10.1111/j.1468-0009.2009.00555.x
- Basu et al., 2013: **The Relationship of Sugar to Population-Level Diabetes Prevalence: An Econometric Analysis of Repeated Cross-Sectional Data** https://doi.org/10.1371/journal.pone.0057873
  "Using econometric models of repeated cross-sectional data on diabetes and nutritional components of food from 175 countries, we found that every 150 kcal/person/day increase in sugar availability (about one can of soda/day) was associated with increased diabetes prevalence by 1.1% (p <0.001) after testing for potential selection biases and controlling for other food types (including fibres, meats, fruits, oils, cereals), total calories, overweight and obesity, period-effects, and several socioeconomic variables such as aging, urbanization and income. No other food types yielded significant individual associations with diabetes prevalence after controlling for obesity and other confounders. The impact of sugar on diabetes was independent of sedentary behaviour"
- NCD-RisC, 2019. **Rising rural body-mass index is the main driver of the global obesity epidemic in adults**. https://www.nature.com/articles/s41586-019-1171-x
  Urban built environment factors? The gap of BMI between urban and rural is closing, mostly by an unprecedented increase in **rural** BMI across the globe in recent years

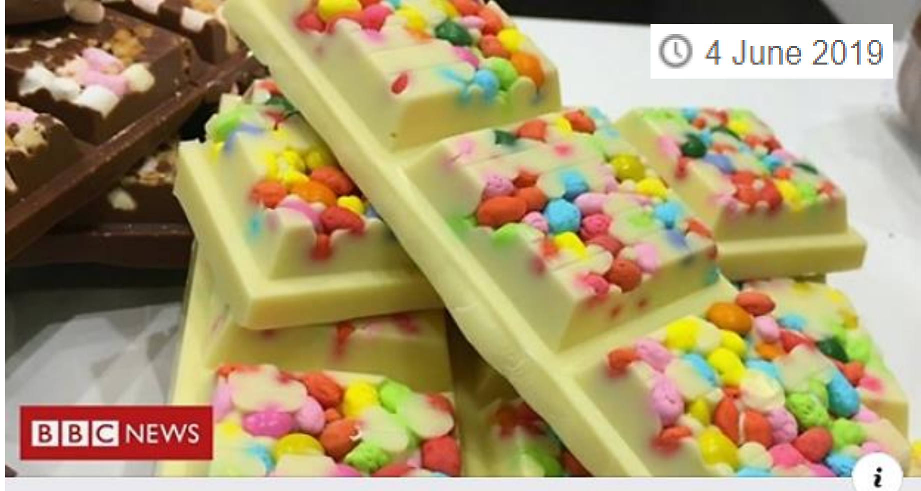

BBC.COM

Is it time to treat sugar like smoking?

https://www.bbc.com/news/health-48499195

Forbes

946,440 views | Apr 24, 2015, 10:44am

Exercise Can't Save Us: Our Sugar Intake Is The Real Culprit, Say Experts

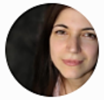

Alice G. Walton Contributor
Pharma & Healthcare

In a fascinating and scorching editorial in the *British Journal of Sports Medicine*, three authors argue that the myth that exercise is the key to weight loss – and to health – is erroneous and pervasive, and that it must end. The evidence that diet matters more than exercise is now overwhelming, they write, and has got to be heeded: We can exercise to the moon and back but still be fat for all the sugar and carbs we consume. And perhaps even more jarring is that

Precision and accuracy public health: community-level physical activity (PA) indicators obtained from population app aggregates

- **Informed public health decision-making (precision and accuracy public health):** In the future, the ideal PA planning and tracking app(s) would expose an API (application programming interface) with suitable privacy provisions/guarantees, policies and data sharing arrangements to enable public health authorities to obtain population aggregates of PA levels and trends over time amongst their target populations (**indicators of community physical activity level/status**), e.g., average steps (or, if Google Fit is used, 'Move Minutes' and 'Heart Points', per person/per day in different age groups and country regions/city neighbourhoods; cumulative steps/ Move Minutes and Heart Points per region/neighbourhood for a given period (can be monitored and compared every few months; can also be normalised by population number to compare different regions/neighbourhoods). This feature should help public health authorities devise superior interventions, better target them and monitor their effect over time, making adjustments to the interventions as necessary.
- (In population aggregates, we are looking for crude trends over time (e.g., overall population PA levels, and whether they are increasing or decreasing or remaining unchanged over time/in different seasons of the year, etc.), so the inaccuracies and differences between different sensors and device models shouldn't pose a big issue, and over- and underestimates will offset each other in large samples.)
- cf. https://www.washingtonpost.com/news/to-your-health/wp/2016/07/15/pokemon-go-leading-to-a-population-level-surge-in-fitness-tracker-step-counts/ and https://watchaware.com/post/17592/cardiogram-users-exercise-more-with-release-of-pokemon-go

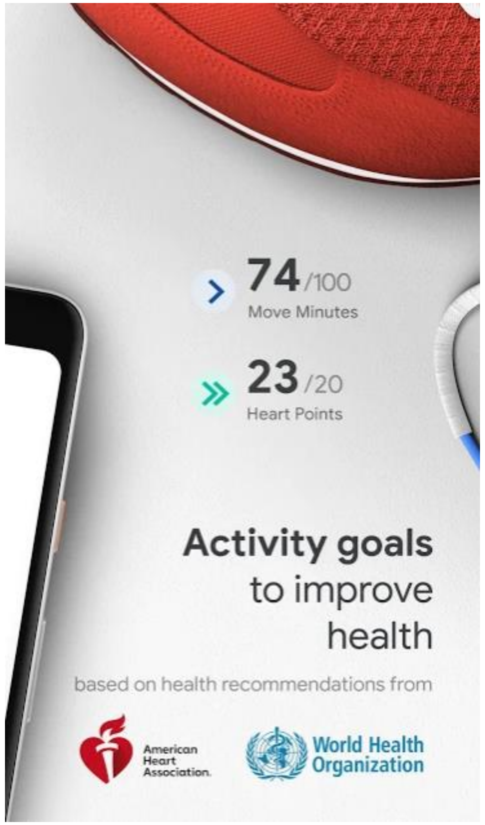

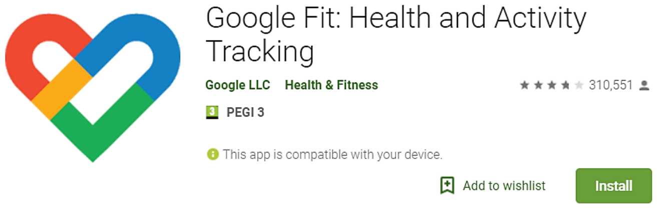

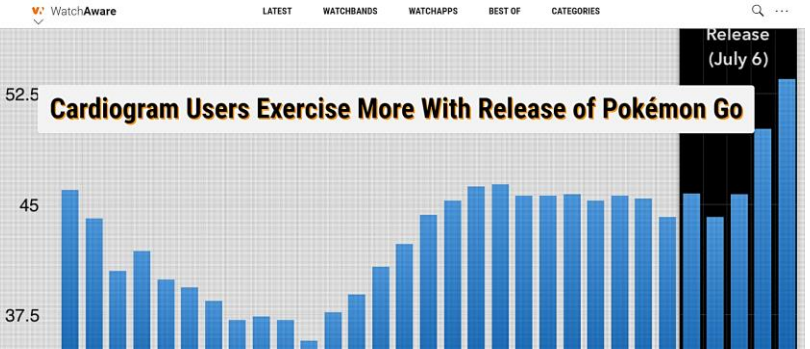

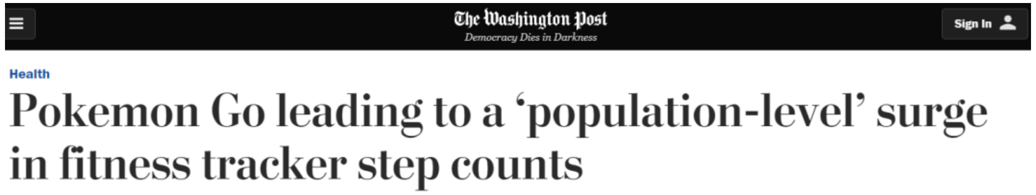

Indicators of community PA status (cont'd)

- Other relevant population-level indicators related to **heart rate variability** (see https://www.health.harvard.edu/blog/heart-rate-variability-new-way-track-well-2017112212789), **sedentary behaviour** and **sleep quality/patterns** in the community can also be extracted from fitness and PA app and gadget big data aggregates of user populations to provide additional 'lifestyle intelligence' for designing better public health interventions.

See: Kamel Boulos and Yang, 2020: **Mobile physical activity planning and tracking: a brief overview of current options and desiderata for future solutions** http://mhealth.amegroups.com/article/view/38687 and Kamel Boulos, 2019: **Towards precision and accuracy digital public health: informed decision-making using novel community-level physical activity indicators from app data aggregates of user populations** https://tinyurl.com/geoweek19

∨ Mi Fit app: heart rate and sleep

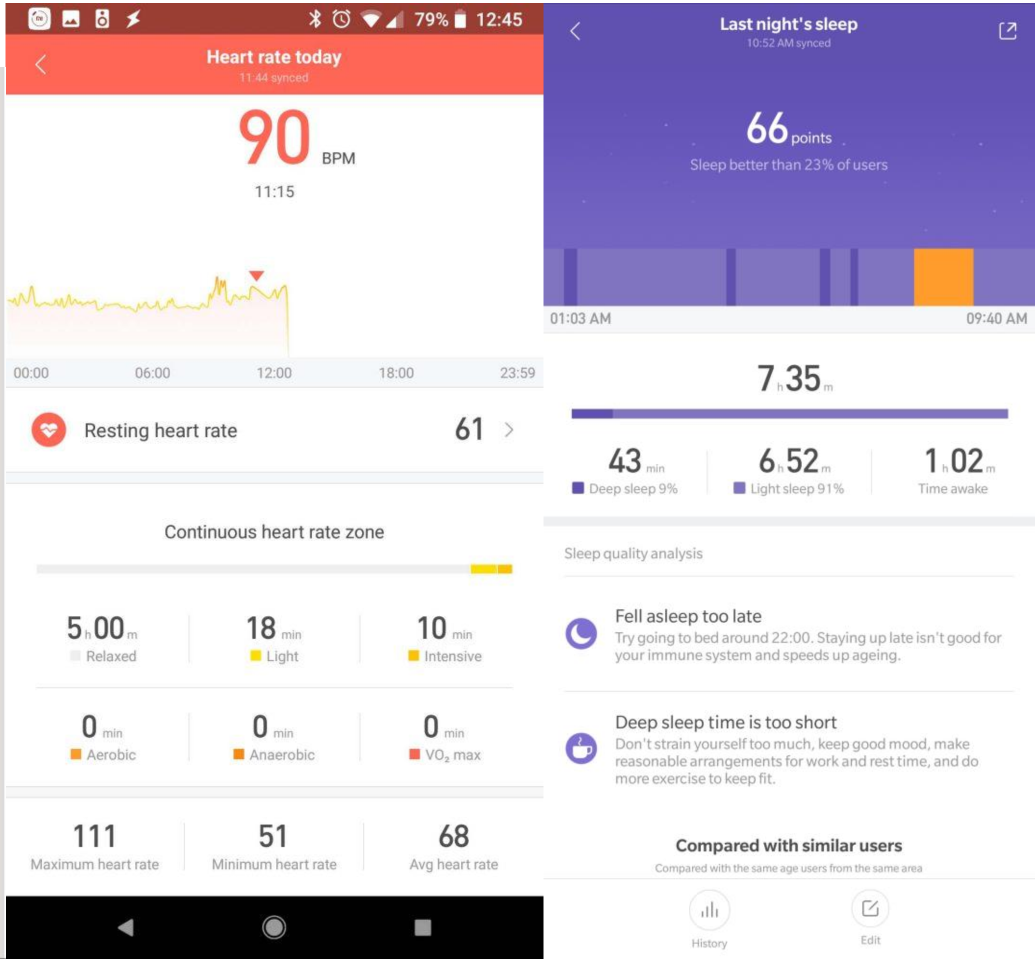

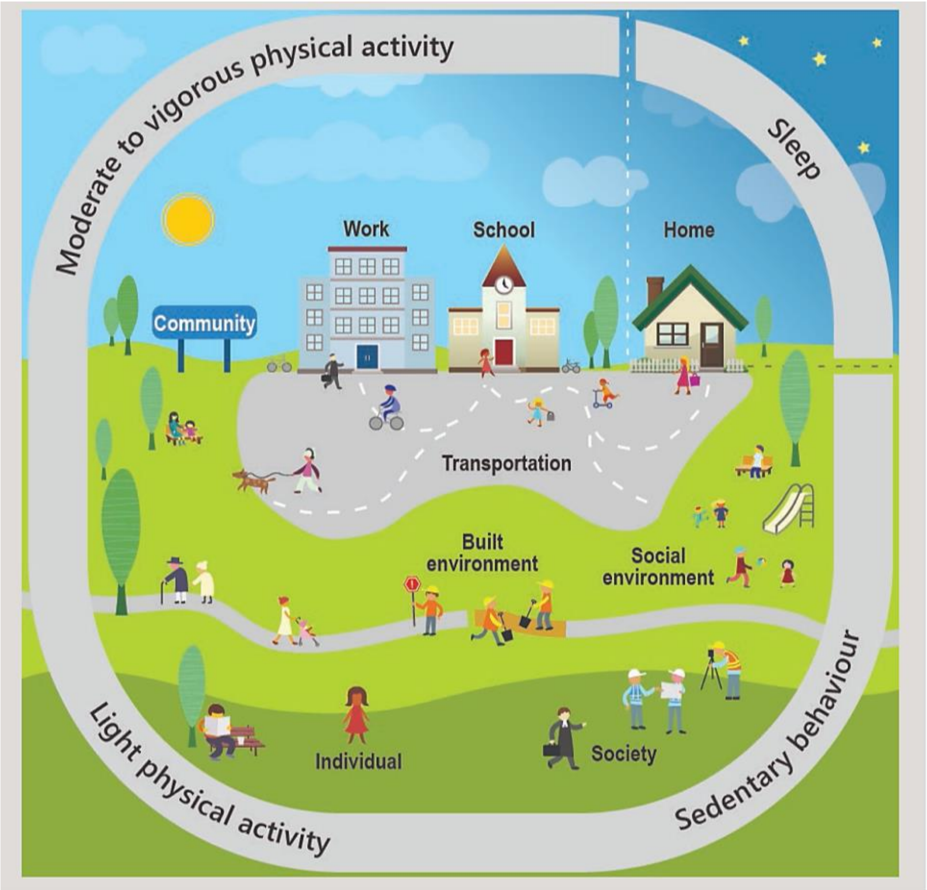

> Roberts KC, Butler G, Branchard B, et al. The Physical Activity, Sedentary Behaviour and Sleep (PASS) Indicator Framework. Health Promot Chronic Dis Prev Can. 2017 Aug;37(8):252-256. doi: 10.24095/hpcdp.37.8.04. (Public Health Agency of Canada)

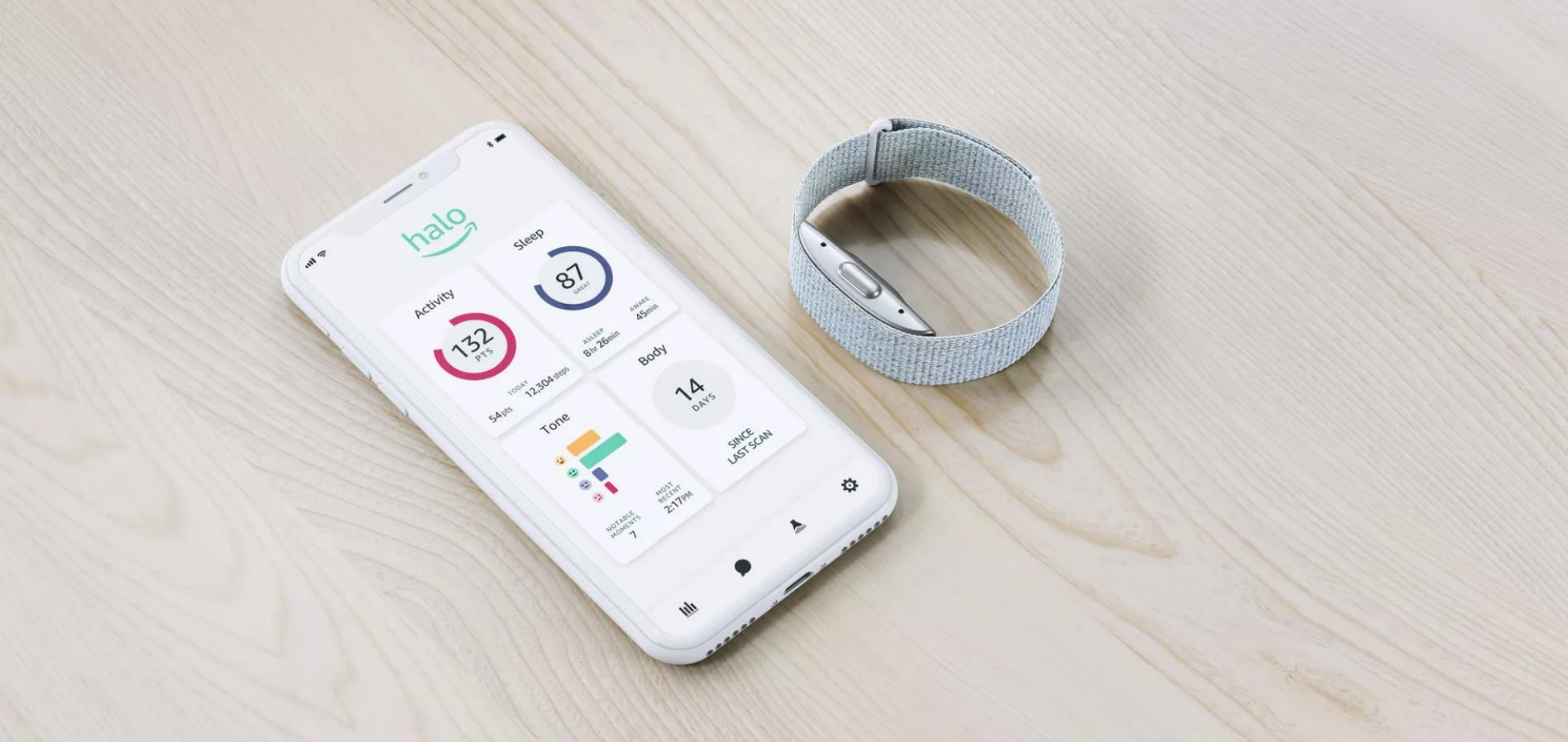

Health and fitness wearable and app data aggregates – emerging possibilities: Amazon Halo subscription service

https://www.aboutamazon.com/news/devices/a-better-measure-of-health

https://www.theverge.com/2020/8/27/21402493/amazon-halo-band-health-fitness-body-scan-tone-emotion-activity-sleep

https://www.theverge.com/2020/8/27/21402493/amazon-halo-band-health-fitness-body-scan-tone-emotion-activity-sleep

Lifestyle data and data sources (Tasks 1 and 2)

- We also need to cover the very latest (and much relevant) published evidence in this area regarding ‘smartphone stress’; the corresponding lifestyle data would be smartphone screen time and usage/app patterns and their relation to harmful cortisol levels spikes and fluctuations throughout the day,* which can precipitate or exacerbate diabetes in the long run

  Data sources: see, for example, TalkingData (a Chinese company) https://www.talkingdata.com/. They offer regularly updated big data-sets of mobile user data and behaviour covering >2.5 billion smart devices in China and more than 200,000 Chinese app developers and their apps

  cf. American users screen time tracking data published by Moment app https://inthemoment.io/. The SMART Study (Canada)** also developed a methodology to derive objective screen-state from population mobile devices (smartphones and others), and found (as reported in one of their unpublished papers – still under peer review as of May 2019) that when objective measures were compared with subjective (self) reporting, the results indicated that participants consistently under-reported screen time

TalkingData

* See, for example, Afifi et al., 2018: WIRED: The impact of media and technology use on stress (cortisol) and inflammation (interleukin IL-6) in fast paced families https://www.sciencedirect.com/science/article/pii/S0747563217306908

** King et al., 2018: The SMART Study, a Mobile Health and Citizen Science Methodological Platform for Active Living Surveillance, Integrated Knowledge Translation, and Policy Interventions: Longitudinal Study https://www.ncbi.nlm.nih.gov/pmc/articles/PMC5893892/

Bluetooth beacons (geolocation) + apps for foot traffic and food environment monitoring—but not without privacy issues

- https://www.nytimes.com/interactive/2019/06/14/opinion/bluetooth-wireless-tracking-privacy.html
- http://www.navi-guide.com/

Most people aren't aware they are being watched with beacons, but the "beacosystem" tracks millions of people every day. Beacons are placed at airports, malls, subways, buses, taxis, sporting arenas, gyms, hotels, hospitals, music festivals, cinemas and museums, and even on billboards.

In order to track you or trigger an action like a coupon or message to your phone, companies need you to install an app on your phone that will recognize the beacon in the store. Retailers (like Target and Walmart) that use Bluetooth beacons typically build tracking into their own apps. But retailers want to make sure most of their customers can be tracked — not just the ones that download their own particular app.

So a hidden industry of third-party location-marketing firms has proliferated in response. These companies take their beacon tracking code and bundle it into a toolkit developers can use.

The makers of many popular apps, such as those for news or weather updates, insert these toolkits into their apps. They might be paid by the beacon companies or receive other benefits, like detailed reports on their users.

Location data companies often collect additional data provided by apps. A location company called Pulsate, for example, encourages app developers to pass them customer email addresses and names.

Companies like Reveal Mobile collect data from software development kits inside hundreds of frequently used apps. In the United States, another company, inMarket, covers 38 percent of millennial moms and about one-quarter of all smartphones, and tracks 50 million people each month. Other players have similar reach.

Location data companies have other disturbing tricks up their sleeve. For example, inMarket developed "mindset targeting" techniques that predict when individuals are most receptive to ads. These techniques are based on statistical probabilities calculated through millions of observations of human behavior.

To protect yourself from beacons in the short term, you can delete any apps that may be spying on you — including apps from retailers — and shut off location services and Bluetooth where they are not needed.

Most of our concerns about privacy are tied to the online world, and can feel theoretical at times. But there is nothing theoretical about Bluetooth beacon technology that follows you into retail stores (and other venues) and tracks your movement down to the meter.

®

Apple and Google could be tracking you through iOS and Android, but they don't make their Bluetooth beacon collection methods transparent. There is no easy way to determine which apps on your phone have the beacon location tracking built in.

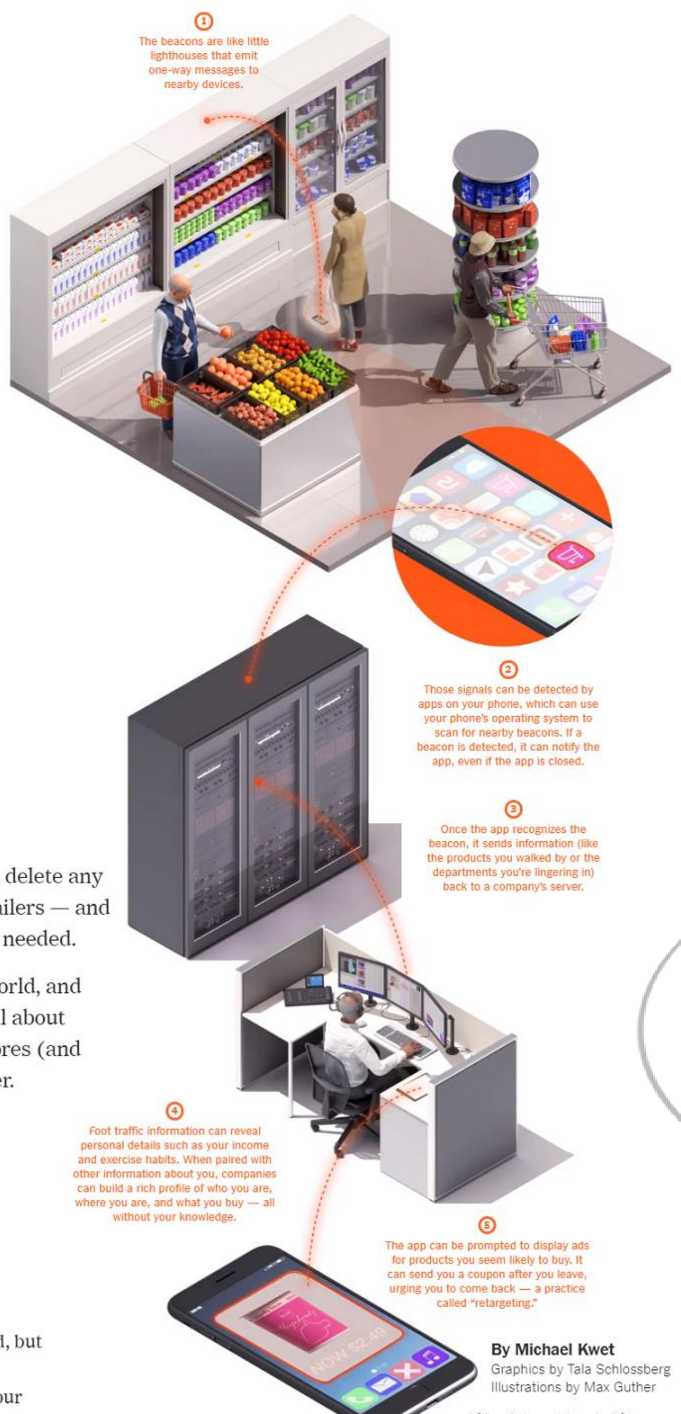

By Michael Kwet
Graphics by Tala Schlossberg
Illustrations by Max Guther
The New York Times

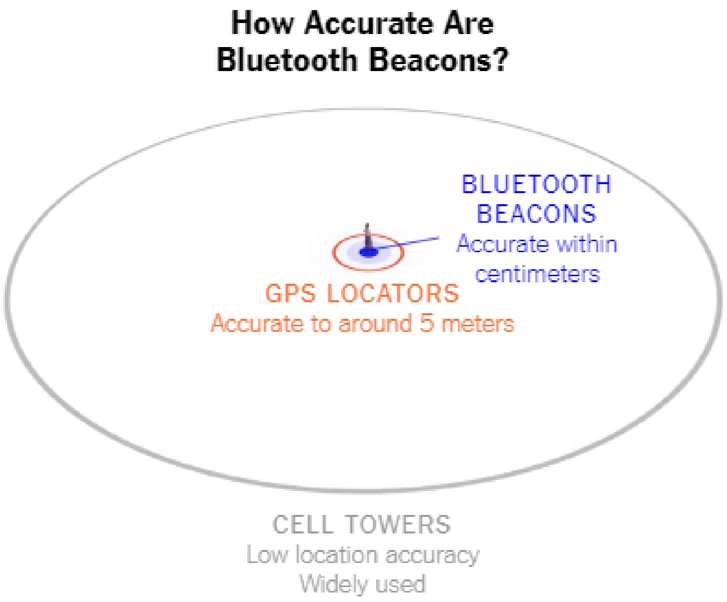

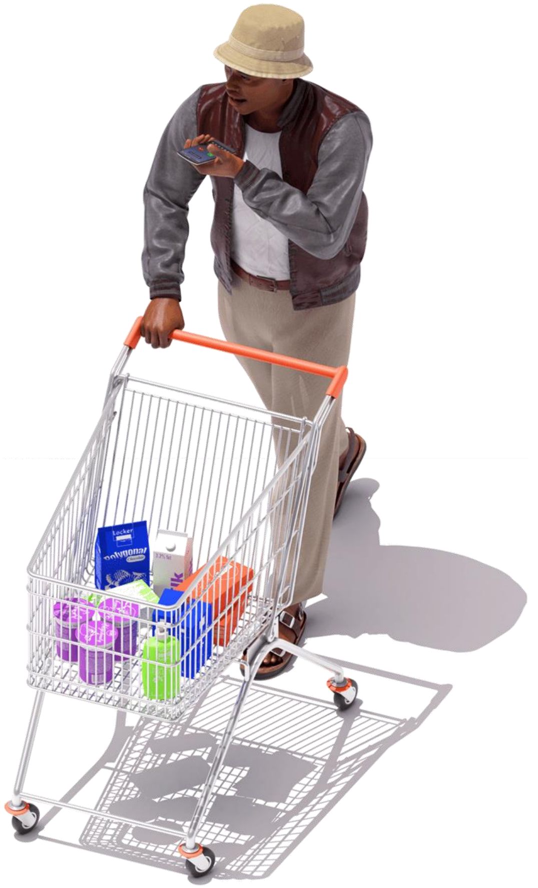

Lifestyle data and data sources (Tasks 1 and 2)

- Proxy mental health data for city population stress/anxiety/depression levels – avg. by age group, gender, neighbourhood, etc.: important factor in overweight/obesity (OO) and binge eating
- Sleep duration and quality data – avg. by age group, neighbourhood, etc. (aggregate city population data from fitness/sleep trackers and apps – if available by special agreements with providers or via a purpose-built campaign/consumer health portal or surveys): poor sleep is a major factor in OO and T2D
- Health literacy levels data – avg. by age group, gender, neighbourhood, etc. (obtained from population surveys or representative samples); media consumption data by media type (different online and social media, different TV stations/programmes, different newspapers and other print material, etc.), by age group, etc.: important to consider when designing mass health education interventions/public health campaigns
- Population clinical data aggregates (anonymised, geo-tagged/aggregated at postcode level or similar, by age group, etc.), updated, say, every 6 months, from clinical screening and other lab tests that are routinely done in hospitals and clinics in our chosen demonstrator location(s) (e.g., the city of Guangzhou), such as BMI, HbA1c, fasting blood glucose, blood pressure, total cholesterol, etc.

  Standards-compliant data linkages/interfaces to electronic hospital/health records can be established to automate the process of collecting these data aggregates (*cf.* syndromic surveillance systems)

<u>No two persons are the same</u>, but there are more finely granular, detailed trends to be learned from <u>population</u> data aggregates and acted upon

- No two persons are the same in how their bodies are affected by, and respond to, <u>exactly the same food environment and diet</u>. We have unique individual profiles (metabolic, genetic, gut bacteria, etc.) that determine our <u>different</u> responses

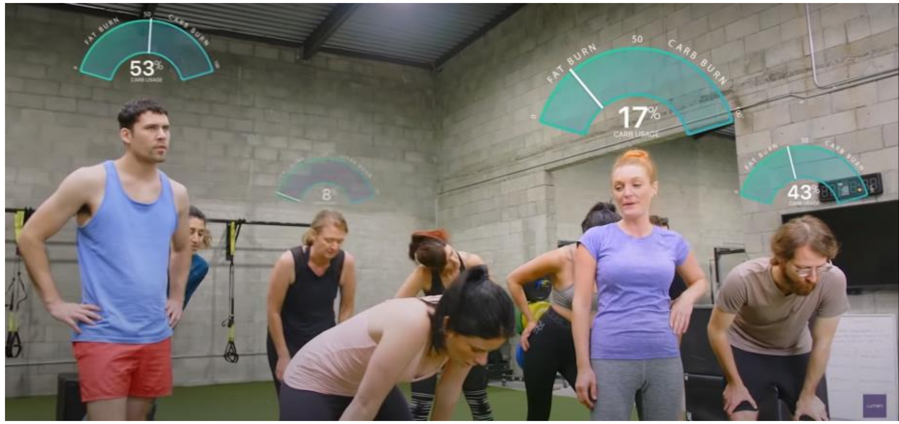

^ Screenshot from https://youtu.be/X8IBgyAMgn4

- In 2015/16, Kamel Boulos proposed a highly personalised diet and lifestyle recommendation system that takes into consideration many of these unique individual profiles using inputs from specialised sensors/wearables, such as mobile personal indirect calorimetry, etc. Besides using users' data aggregates (aggregates of the same user and of all system users over time) for its own dynamic self-learning and tweaking, the system would also offer **a privacy-preserving version of its user population data aggregates for research and public health purposes**

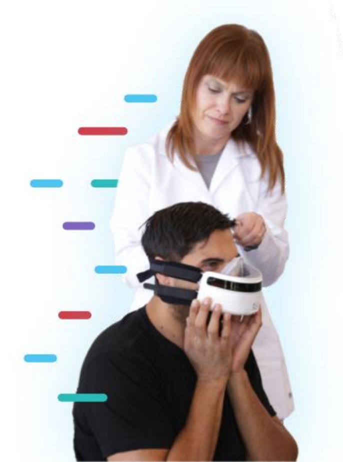

Kamel Boulos MN. ADAMILO—Automated Diet and Activity Monitoring for Intelligent Lifestyle Optimization (Session: eHealth in Primary and Emergency Care, Friday 8 April 2016, 09:00-10:30, Conference Room 4). Full paper in: *Proceedings of Med-e-Tel - The International eHealth, Telemedicine and Health ICT Forum for Education, Networking and Business; an event of the International Society for Telemedicine & eHealth (ISfTeH), Luxembourg, 6-8 April 2016* / Jordanova M, Lievens F (Editors). Global Telemedicine and eHealth Updates: Knowledge Resources. Grimbergen, Belgium: ISfTeH, 2016; 9:71-78 (print edition - ISSN 1998-5509 / CD-ROM: Med-e-Tel Electronic Proceedings 2016, pp.560-567 - ISSN 1818-9334). Abstract only: *JISfTeH - Journal of the International Society for Telemedicine and eHealth* (ISSN 2308-0310) 2016;4:eS1 <URL: http://journals.ukzn.ac.za/index.php/JISfTeH/article/view/162/html#primary>

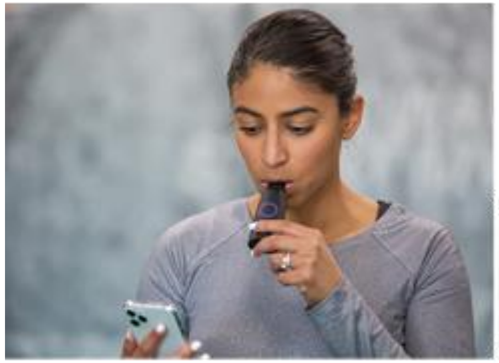

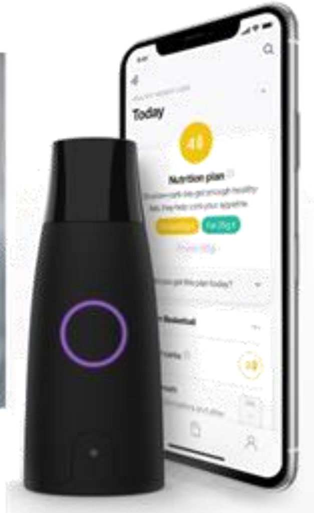

Breezing https://breezing.com/ < mobile indirect calorimetry > https://www.lumen.me/ Lumen

Lifestyle data and data sources (Tasks 1 and 2, if we are now **in 2025**)

Future directions (beyond this demonstrator): factoring in population genetics/epigenetics and gut microbiome profile aggregates from (future) genome data banks and gut microbiome data banks

A calorie is not a calorie:

- Different people with different genetic and gut bacteria profiles absorb and burn exactly the same type and amount of food at different rates. Individual variations also exist in learned lifestyle habits, gut hormones profiles, which control appetite, etc.
- Calories from same food type and amount can vary according to whether the food was left to cool down (less calories, even when reheated) or was consumed fresh and hot (more calories), e.g., toast, potatoes, rice and pasta, due to formation of resistant starch
- etc.

https://youtu.be/avcQy2_yEkk

People's appetite and response to so-called 'obesogenic food environments' vary according to their genetic and epigenetic profiles

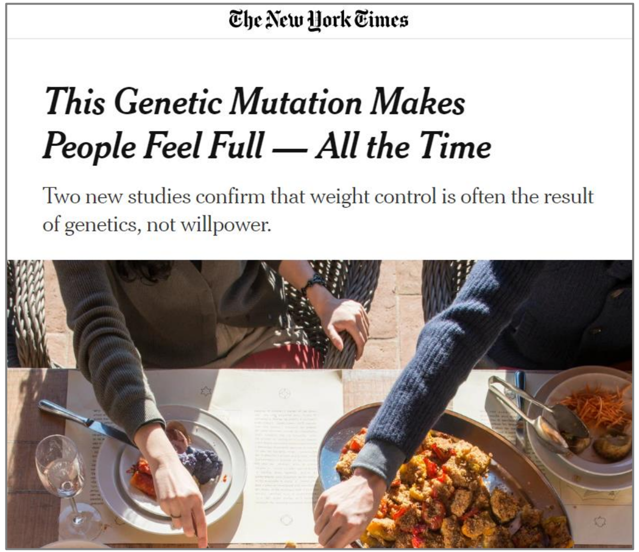

< Lotta et al., 2019: Human Gain-of-Function MC4R Variants Show Signaling Bias and Protect against Obesity https://www.cell.com/cell/fulltext/S0092-8674(19)30345-9

Click to watch video (online) >

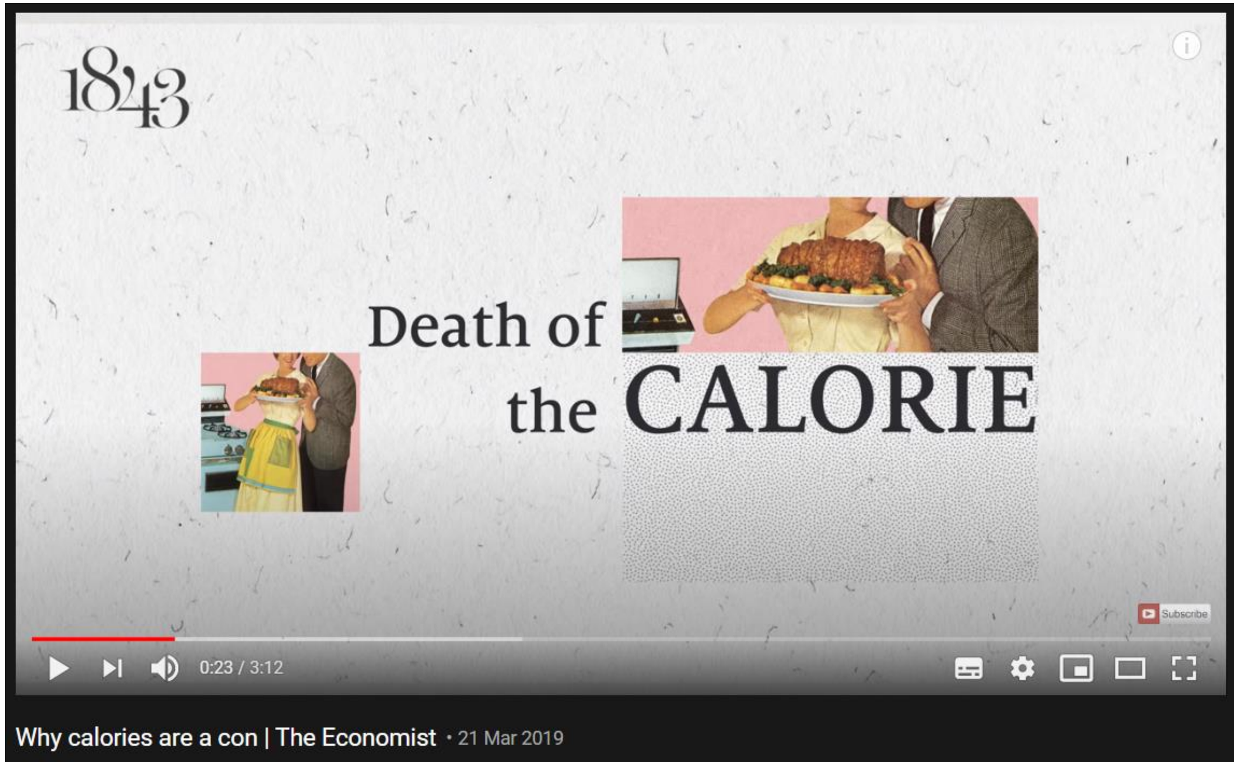

Lifestyle data and data sources (Tasks 1 and 2, if we are now **in 2025**)

Future directions (beyond this demonstrator): factoring in population genetics/epigenetics and gut microbiome profile aggregates from (future) genome data banks and gut microbiome data banks

Same can be said about physical activity:

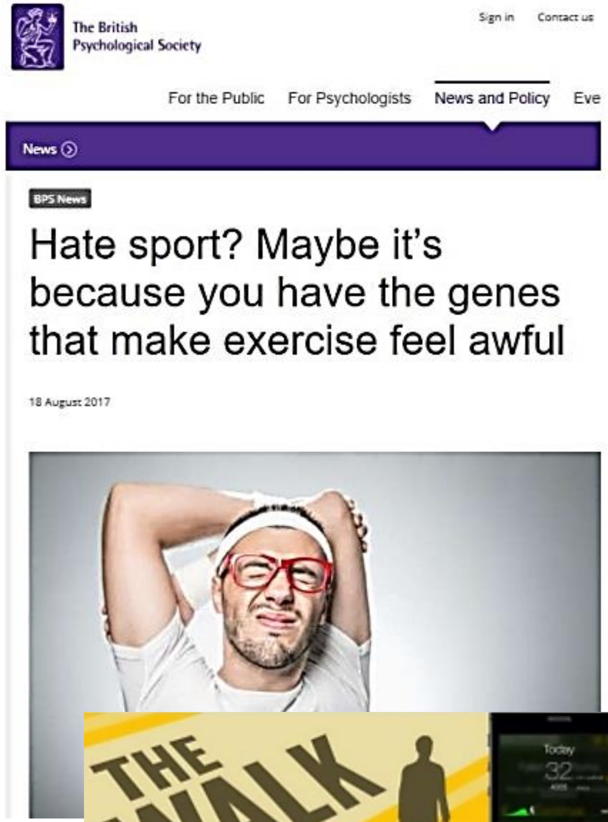

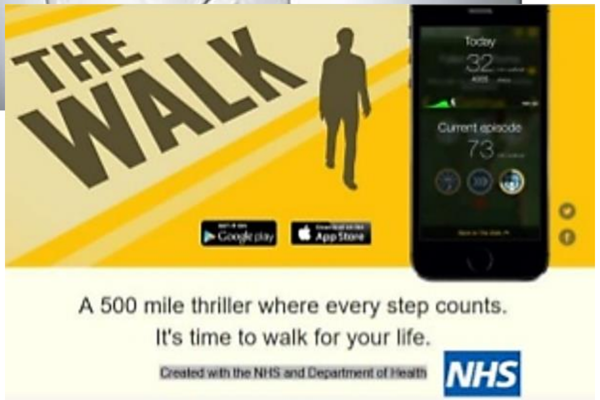

< Schutte NM, Nederend I, Hudziak JJ, Bartels M, de Geus EJC. Heritability of the affective response to exercise and its correlation to exercise behavior. *Psychol Sport Exerc*. 2017;31:139-148. doi: 10.1016/j.psychsport.2016.12.001 — See also: doi: 10.1016/S0140-6736(12)60735-1

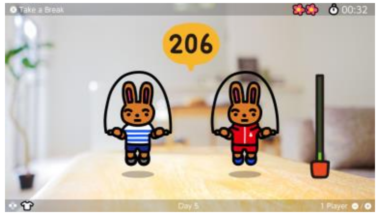

But that's not the end of the story, we can still **'tame' (tone down/turn off expression of) those unfavourable genes** with **suitable external factors/interventions**, e.g., **gamified** exercise interventions (**exergames**) for extra 'appeal' and behavioural sustainability in those individuals that 'hate' exercise because of their genetic makeup

Epigenetics: the study of changes in organisms caused by **modification of gene expression** rather than alteration of the genetic code itself

The **genome/genes** confer potential protections and predispositions, but it is the **lifestyle/environmental (exposome)** modulation, up and down/on and off, of their expression **(gene expression) via the epigenome (epigenetics)** that determines their **ultimate effects, i.e., can increase/enhance or decrease/mute the negative (and positive) effects of lifestyle and environmental exposures on the individual**, depending on the unique interplay between a person's genetic profile and his/her lifestyle/environmental exposures. ***Some*** of the epigenome-tagged genome DNA and histones can even be **heritable** (passed on to offsprings)
For a good intro, see https://www.genome.gov/about-genomics/fact-sheets/Epigenomics-Fact-Sheet

On the complex interplay of environment, genes, and other factors in disease: https://harvardmagazine.com/2019/07/nature-nurture-genetics

> "*It's common to think of disease and health "as this tension of ZIP code* (postcode) *versus genetic code," explains Chirag Patel, assistant professor of biomedical informatics at Harvard Medical School.*
>
> "*But a study by Patel and his research team challenges this "either-or" thinking, using Big Data to tease apart the complex interplay of environment, genes, and other factors in disease.*"

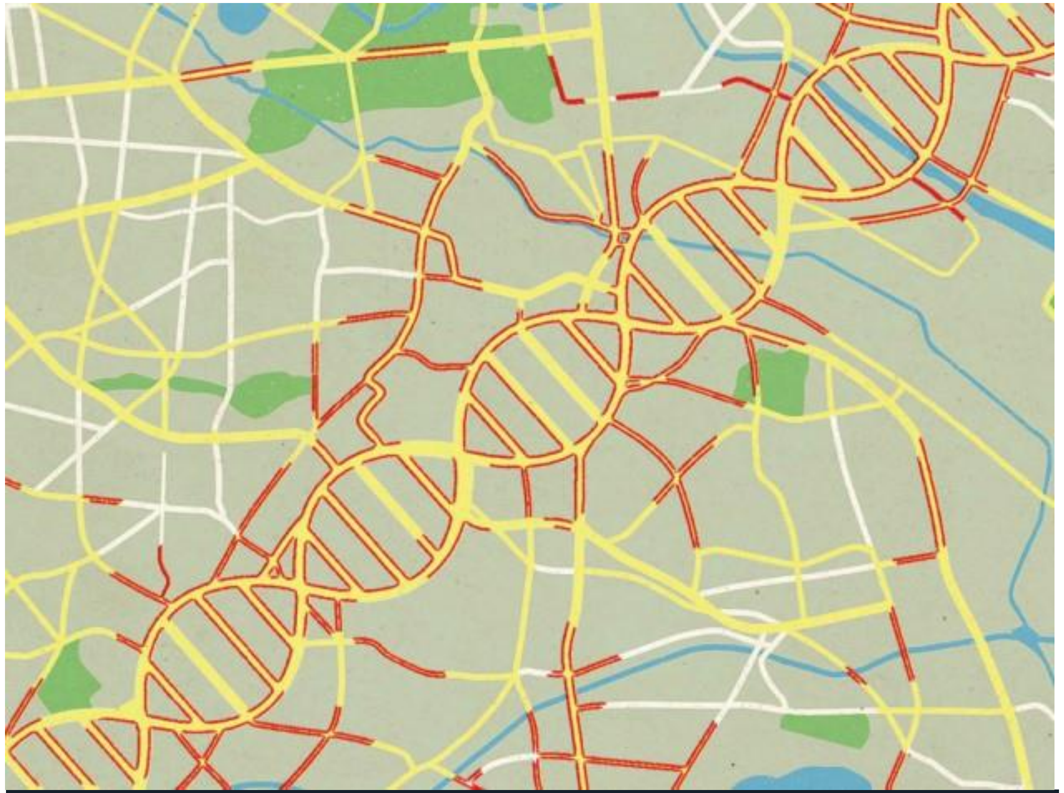

Zip Code vs. Genetic Code

The largest-ever study of twins quantifies the respective influence of genes and environment on specific diseases.

harvardmagazine.com

Association between gut microbiota composition and individual- and area-level socioeconomic factors

- See https://www.ncbi.nlm.nih.gov/pmc/articles/pmid/30641975/
- Even identical twins (same genetic makeup) had different responses to the same foods, which might be down to their **gut microbes** as the twins shared only 37% of the same microbes with each other. This is only slightly more than unrelated individuals, which share an average of 35% of the same microbes

Identical twins have different responses to the same food

Print A A A

June 17, 2019

The first results from the largest ongoing scientific nutrition study of its kind suggest that individual responses to the same foods are unique, even between identical twins. Researchers—led by an international team of leading scientists including researchers from King's College London, Massachusetts General Hospital, and nutritional science company ZOE— presented their findings at the American Society of Nutrition and the American Diabetes Association conferences.

http://www.ift.org/Food-Technology/Daily-News/2019/June/17/identical-twins-have-different-responses-to-the-same-food.aspx

microorganisms MDPI

Microorganisms. 2019 Jan; 7(1): 17. PMCID: PMC6351927
Published online 2019 Jan 11. doi: 10.3390/microorganisms7010017 PMID: 30641975

Socioeconomic Status and the Gut Microbiome: A TwinsUK Cohort Study

Ruth C. E. Bowyer,[1] Matthew A. Jackson,[1,2] Caroline I. Le Roy,[1] Mary Ni Lochlainn,[1,3] Tim D. Spector,[1] Jennifer B. Dowd,[4,5,†] and Claire J. Steves[1,6,*†]

Lifestyle data and data sources (Tasks 1 and 2, if we are now **in 2025**)

Future directions (beyond this demonstrator): factoring in population genetics/epigenetics and gut microbiome profile aggregates from (future) genome data banks and gut microbiome data banks

- In the future, we will know more about our target populations, thanks to **population genome data banks**, where profiles of large local population samples can be mined and analysed for the presence specific/relevant genes/gene variants/mutations and dysfunctions, e.g., those controlling appetite, or implicated in overweight and obesity predisposition, or determining our base exercise behaviour: FTO, LEP, MRAP2, MC4R, SDC3, etc., and use this intelligence to inform the design of optimised individual-specific as well as **population-wide interventions** that can make our genes work best for us and/or offset their negative effects (predispositions)
- Similarly, we can have **population gut microbiome data banks** (with regularly updated profiles, say every 6 months) of large local population samples. A growing number of consumer-oriented labs are offering 'gut bacteria profiling' services today at affordable prices, e.g., DayTwo, Viome, AmericanGut, Aperiomics, AtlasBiomed and others. Gut bacteria types and diversity/ratios (some are 'bad' in relation to obesity, e.g., *Firmicutes*, while others are 'good', e.g., *Christensenella and Bacteroidetes* – see, for example, https://www.ncbi.nlm.nih.gov/pmc/articles/PMC5949328/ and https://www.ncbi.nlm.nih.gov/pmc/articles/PMC5933040/) can be modified or modulated/ influenced, as necessary, through both individual-specific and **population-wide interventions** (dietary modifications/mass health education about diet) to make our gut microbiomes work best for us (e.g., **encourage the consumption of sauerkraut (cabbage), yoghurt, kimchi and miso soup, all of which promote good gut bacteria**)
- **Future challenges** associated with the above data banks: protection of genomic and other sensitive population data from being used against employment and health insurance, and informed consent on storing and using genetic and nongenetic information for research and development (see https://www.ncbi.nlm.nih.gov/pmc/articles/PMC4915347/)

Lifestyle data and data sources (Tasks 1 and 2, if we are now **in 2025**)

A new era of Accuracy and Precision Public Health and Medicine

- **Population-level accuracy and precision public health interventions:** Only if we have evidence of a large proportion of the population with, say, the wrong gut bacteria profiles (bacteria that promote obesity), then we can roll-out some targeted health education and mass diet interventions to rectify this. Such population evidence will be drawn from 'gut microbiome banks' that store and regularly update the gut bacteria profiles of very large numbers of individuals (numbers that are sufficient to be used as a crude proxy for the whole population). Such gut microbiome data banks do not yet exist, so can be ruled out as a data source in the proposed demonstrator. Same can be said about genetic profiles banks until we have them one day
- This does not rule out general (non-targeted) whole-population interventions with respect to these factors, as long as their cost/benefit ratio is reasonable (not always the case), e.g., general health education and mass dietary interventions to promote good gut bacteria or gamified exercise interventions or exergames (not just for those individuals that 'hate' exercise because of their genetic makeup). Such interventions would still benefit the rest of the population in varying degrees; for example, people who already enjoy exercising and have adequate daily exercise levels might still like and benefit from exergames, etc.
- But such accuracy and precision public health interventions, for our purposes in this demonstrator, cannot be seen as targeted (as a response to specific population data/intelligence we have) and their immediate effects cannot be easily monitored (we don't have, for example, a gut microbiome bank that we can use to show and document a significant change in population gut microbiome profiles following an intervention of this type)
- **On an individual (person-specific) level, similar (accuracy and) precision medicine interventions** are already being prescribed today based on findings from the person's specific genetic makeup and gut bacteria profile

Conclusion: The research we should be doing

Towards precision and accuracy health/public health

- The segmentation of food consumption in large populations (per capita) by food type, and the associations of this segmentation/segmentation patterns with different population characteristics and of course overweight and obesity, remain an important research gap in the current literature. It is the type (and relative quantities) of diet that matters the most in overweight and obesity, and not just the total number of calories consumed, the amount of physical activity undertaken, or the proximity to supermarkets or KFCs. The same food store, e.g., a supermarket or KFC, can often sell/serve good and bad calories. One hundred (100) calories from a 250ml sugary beverage and 100 calories from a handful of nuts are not the same. The segmentation of calories is essential, as not all calories have equal effect with respect to obesity, or as they say 'a calorie is not a calorie'. The type of food outlet/food type sales proportions per outlet, e.g., outlets selling (and proportion of sales of) unprocessed vs. highly processed food; the specific types of food bought by different households as determined by their socioeconomic levels (supermarket loyalty cards are one possible data source here); and even screen time (average duration per capita)/screen time patterns, especially in children, etc., are the factors that really matter in childhood and adult obesity, and the sort of detail resolution we should be investigating in future studies

- Similarly, when it comes to physical activity (PA) tracking in populations and PA facilities and opportunities in the neighbourhood environment, they should also be segmented by type of activity, taking into consideration the different genetic profiles of population subgroups. A recent study (**https://doi.org/10.1371/journal.pgen.1008277**) found that regular jogging, followed by mountain climbing, walking, power walking, certain types of dancing, and long yoga practices reduced BMI in individuals who are genetically predisposed to obesity. But cycling, stretching exercises, swimming and Dance Dance Revolution (an exergame) did **not** counteract the genetic effects on obesity! If we want to deliver effective and truly individualised PA advice and coaching, we have to include this sort of detail resolution about the opportunities and uptake of different PA types by different population subgroups in the neighbourhood

Population dietary behaviours, including amounts consumed per individual snack/meal/day or per family are also important, as even the healthiest options can prove unhealthy when overconsumed!

Performing different kinds of physical exercise differentially attenuates the genetic effects on obesity measures: Evidence from 18,424 Taiwan Biobank participants

Wan-Yu Lin, Chang-Chuan Chan, Yu-Li Liu, Albert C. Yang, Shih-Jen Tsai, Po-Hsiu Kuo

Published: August 1, 2019 • https://doi.org/10.1371/journal.pgen.1008277

Swimming in cold water may stimulate appetite and prevent weight loss, warns scientist

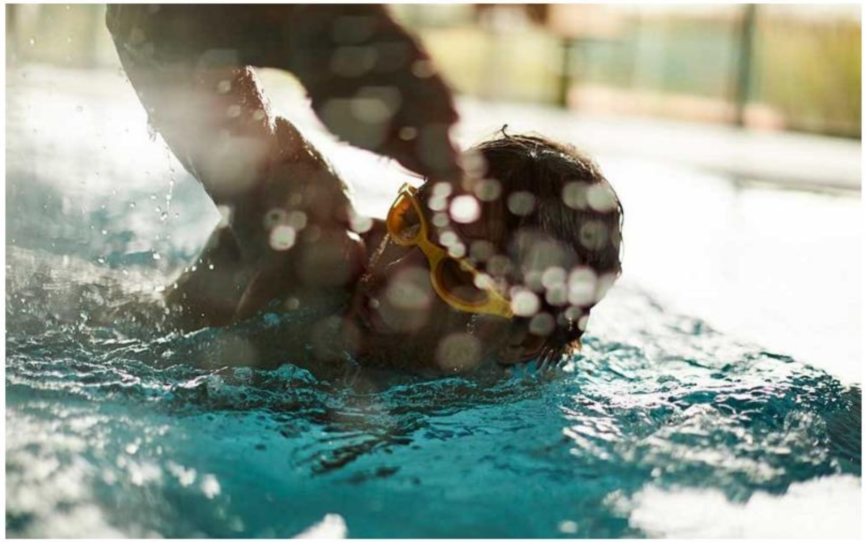

Swimming may be a pointless exercise for people with obesity genes, the University of Taiwan believes. CREDIT: ALAMY

^ https://www.telegraph.co.uk/science/2019/08/01/swimming-cold-water-may-stimulate-appetite-prevent-weight-loss/

Cauda: Moving from reactive to predictive and proactive public health

- A **smart (healthy) city** should continuously monitor and sense the state (or '**pulse**') of its population and respond in real or near-real-time to any flagged needs or unfolding or predicted problems in efficient and effective manners (see http://biomedcentral.com/collections/smarthealthycities)
- Our approach in this demonstrator is similar to the one already used in digital syndromic surveillance (since the early 2000s) to detect early/emerging outbreaks and bioterrorist attacks in the community in real-or near real time using crude population data, but our public health goals and data sources will be different:
  Whilst syndromic surveillance relies on real or near-real-time geo-tagged big data (city, region/province or state/country level) about OTC (over-the-counter) drug sales, weather data, outpatient/GP clinics patient summaries, etc., our lifestyle big data collection will come from different geo-tagged sources, e.g., mobile apps/mobile crowdsensing/smartphone sensor data, telecom data (population daily trajectories/mobility data), various retailers' data, online/social media streams, different government (census, demographic and socio-economic, etc.) and hospital data sources, various surveys,* map data (base map, land use and Street View data), including lifestyle POIs (points of interests), such as restaurants, tobacco/alcohol outlets, etc. Appropriate analytics and GIS (including GeoAI) methods will be used to merge and reason with these data in near real-time to provide strategic health intelligence, with the view of better informing public health policy makers/programme directors and helping them prioritise and better design/target/optimise their public health interventions and efforts for those parts and districts of a city or region/population groups that are most in need
- This could also reduce the costs of such population-level interventions (by better [**more accurately and precisely**] targeting them to only those in need) and **maximise the return value of every RMB (CN¥) spent on public health**

* Surveys: Questionnaires/self-reporting diaries – can suffer from reliability/recall accuracy issues (under-reporting), in addition to slow and costly updates, being largely manual instruments; these issues should be carefully taken into account when agreeing on and designing our final data sources
For example: "*Self-reported time spent walking and biking was strongly overestimated when compared to GPS measurements*" (Klous et al., 2017)
"*To accurately assess children's active transport in leisure time, GPS measures are recommended over self-reports*" (Vanwolleghem et al., 2016)
Same has been shown in the peer reviewed literature about food diaries/questionnaires

Additional bibliography (besides those presented throughout this slide set) - 1

- Kamel Boulos et al., 2021: **Smart city lifestyle sensing, big data, geo-analytics and intelligence for smarter public health decision-making in overweight, obesity and type 2 diabetes prevention: The research we should be doing** (in *International Journal of Health Geographics*, vol. 20)
- Liu Y et al., 2019: **The linkage between the perception of neighbourhood and physical activity in Guangzhou, China: Using street view imagery with deep learning techniques** https://ij-healthgeographics.biomedcentral.com/articles/10.1186/s12942-019-0182-z
- Präger et al., 2019, in press (IJHG): **Using data from online geocoding services for the assessment of environmental obesogenic factors: A feasibility study**
- Zhao, Kwan and Zhou, 2018: **The Uncertain Geographic Context Problem in the Analysis of the Relationships between Obesity and the Built Environment in Guangzhou** https://www.mdpi.com/1660-4601/15/2/308
- Kamel Boulos and Le Blond, 2016: **On the road to personalised and precision geomedicine: medical geology and a renewed call for interdisciplinarity** https://ij-healthgeographics.biomedcentral.com/articles/10.1186/s12942-016-0033-0
- Liu Tongtong et al., 2018: **Temporal understanding of human mobility: A multi-time scale analysis** https://www.ncbi.nlm.nih.gov/pmc/articles/PMC6258540/
- Alessandretti et al., 2017: **Multi-scale spatio-temporal analysis of human mobility** https://www.ncbi.nlm.nih.gov/pmc/articles/PMC5310761/
- Williams et al., 2015: **Measures of Human Mobility Using Mobile Phone Records Enhanced with GIS Data** https://www.ncbi.nlm.nih.gov/pmc/articles/PMC4507852/
- Kang et al., 2018: **Capturing fine-scale travel behaviors: a comparative analysis between personal activity location measurement system (PALMS) and travel diary** https://www.ncbi.nlm.nih.gov/pubmed/30509275
- Ruktanonchai et al., 2018 (IJHG): **Using Google Location History data to quantify fine-scale human mobility** https://www.ncbi.nlm.nih.gov/pubmed/30049275
- Van Hecke et al., 2018: **Factors related with public open space use among adolescents: a study using GPS and accelerometers** https://www.ncbi.nlm.nih.gov/pmc/articles/PMC5778634/
- McCrorie et al., 2014: **Combining GPS, GIS, and accelerometry to explore the physical activity and environment relationship in children and young people – a review** https://www.ncbi.nlm.nih.gov/pmc/articles/PMC4172984/

Additional bibliography (besides those presented throughout this slide set) - 2

- Jankowska et al., 2015: **A Framework For Using GPS Data In Physical Activity And Sedentary Behavior Studies** https://www.ncbi.nlm.nih.gov/pmc/articles/PMC4272622/
- **Beyond the 'energy imbalance (calories in, calories out)' model**-- Ebbeling, Ludwig et al., 2018 (BMJ): **Effects of a low carbohydrate diet on energy expenditure during weight loss maintenance: randomized trial** https://www.ncbi.nlm.nih.gov/pmc/articles/PMC6233655/
- Pantell et al., 2019: **Association of Social and Behavioral Risk Factors With Earlier Onset of Adult Hypertension and Diabetes** https://jamanetwork.com/journals/jamanetworkopen/fullarticle/2733431
- PubMed has many papers on **WHO Healthy Cities** networks/topics, including some by Kamel Boulos et al. (2014 onwards); some articles are from the previous (20th) century; note how the concept of '**smart**' is a relatively recent addition: https://www.ncbi.nlm.nih.gov/pubmed/?term=WHO+%22healthy+cities%22
  Kamel Boulos and WHO colleagues, 2014/2015: https://www.ncbi.nlm.nih.gov/pmc/articles/PMC4417221/ and https://www.ncbi.nlm.nih.gov/pmc/articles/PMC3987056/
- Say 'smart **regions**', not (just) 'smart cities': Jakarta statement – note how villages, towns and islands (aka regions) are now explicitly mentioned in addition to cities proper: https://www.who.int/healthpromotion/conferences/previous/jakarta/statements/healthy_cities/en/
  WHO Healthy Cities EU network: http://www.euro.who.int/en/health-topics/environment-and-health/urban-health/who-european-healthy-cities-network - Asia and other parts of the world have their regional WHO Healthy Cities networks
- Kamel Boulos et al., 2011: **Crowdsourcing, citizen sensing and sensor web technologies for public and environmental health surveillance and crisis management: trends, OGC standards and application examples** https://www.ncbi.nlm.nih.gov/pmc/articles/PMC3271966/
- Kamel Boulos, 2013 (GEOMED 2013): **Public engagement and participation in health geography: crowdmaps (crowdsourced maps) by citizens, for citizens** https://www.slideshare.net/sl.medic/public-engagement-and-participation-in-health-geography
- Mathys and Kamel Boulos, 2011: **Geospatial resources for supporting data standards, guidance and best practice in health informatics** https://www.ncbi.nlm.nih.gov/pmc/articles/PMC3224535/
- A relevant EU H2020 project to learn from (2016-April 2020; budget: 5 Million Euros): http://www.project-pulse.eu/ / http://www.project-pulse.eu/about/objectives/ "**The PULSE—Participatory Urban Living for Sustainable Environments project** will pioneer the development and testing of dynamic spatio-temporal health impact assessments using geolocated population-based data"
  See paper: **Spatial Enablement to Support Environmental, Demographic, Socioeconomics, and Health Data Integration and Analysis for Big Cities** https://www.frontiersin.org/articles/10.3389/fmed.2019.00084/full
